# Solvent-dependent metabolomic profiling of *Acacia mearnsii* bark: an untargeted metabolomics approach

**DOI:** 10.1007/s00216-026-06496-0

**Published:** 2026-04-29

**Authors:** Carolina Feistauer Gomes, Giovana Domeneghini Mercali, Eliseu Rodrigues

**Affiliations:** Institute of Food Science and Technology, Federal University of Rio Grande do Sul (UFRGS), Avenida Bento Gonçalves, 9500, Porto Alegre, RS 91509-900 Brazil

**Keywords:** Metabolites, Proanthocyanidins, Extraction solvents, Food additive, Feed development

## Abstract

With great social and economic importance, black wattle bark (*Acacia mearnsii* De Wild.) stands out due to its high content of condensed tannins. Although it is a known and exploited source of proanthocyanidins, little is known about the other compounds that may be present in this raw material and that may further contribute to its bioactive properties. Given this context, this work focused on applying untargeted metabolomics to investigate the metabolic diversity of black wattle bark using different extraction solvents. Furthermore, it aimed to investigate the proanthocyanidin profile of the raw material cultivated in southern Brazil. It was observed that the use of different solvents influenced the extraction and composition of metabolites, with the main differences observed between aqueous and organic solvents. Thus, 50% and 70% acetone, as well as 70% methanol, achieved the highest relative abundances in terms of metabolites. Eighty different compounds were annotated, including organic acids and phenolic compounds, among others, many of which were detected for the first time in black wattle bark. These results demonstrate the richness of the extracts in terms of their metabolic composition, exhibiting chemical complexity far beyond the condensed tannins. Regarding proanthocyanidins, the presence of oligomers up to pentamers was observed. The monomers observed were varied, with (epi)catechin and (epi)gallocatechin acting as polymer chain initiators, while (epi)robinetifinol and fisetinidol are the extender units. The metabolic diversity verified in black wattle bark justifies the potential use of its extracts as natural additives to be explored by the food and feed industries.

**Graphical abstract:**

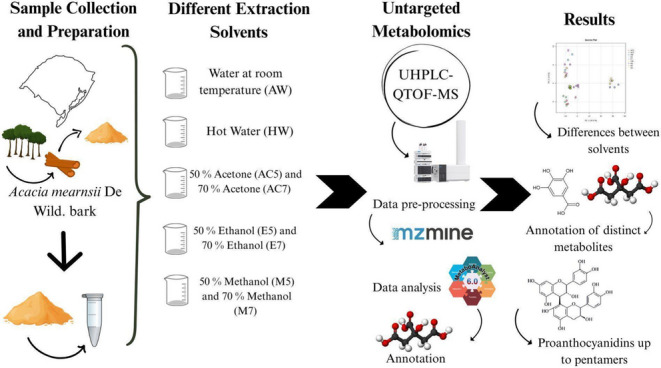

**Supplementary Information:**

The online version contains supplementary material available at 10.1007/s00216-026-06496-0.

## Introduction

Black wattle (*Acacia mearnsii* De Wild.) is a leguminous tree with significant economic and social importance. Native to Australia, it is currently mainly cultivated in southern Brazil and South Africa, being the only temperate species of *Acacia* commercially planted on a significant international scale [1, 2]. The bark of black wattle yields a tannin-rich extract, traditionally used in the leather industry due to its ability to bind to proteins [3].

Tannins belong to the diverse group of phenolic compounds and are widely distributed in nature. These secondary metabolites are produced by plants under stressful conditions, playing a protective role in plant defense mechanisms. Tannins are compounds with molecular masses ranging from 500 to 20,000 Da, and their chemical structure varies widely [4]. Among the various tannin groups, proanthocyanidins (or condensed tannins) stand out due to their significance across diverse industrial applications. Recently, tannins derived from wood and tree bark have gained attention for their novel and promising applications in the food sector and other industries [5].

Although *Acacia mearnsii* De Wild. bark is mainly recognized for its high proanthocyanidin content, little is known about the other compounds found in this raw material, which may also contribute to the biological and technological activities attributed to their extracts. Kusano et al. [6] reported the presence of other phenolic compounds (in addition to proanthocyanidins) in black wattle bark, including taxifolin, butin, and syringic acid, among others. However, its composition may be much more complex, extending beyond polyphenols, a topic that remains largely unexplored in scientific literature. In this context, untargeted metabolomics emerges as a valuable tool to investigate the diverse composition of tannin-rich extracts from black wattle bark.

In untargeted metabolomics, the choice of extraction method is crucial, as it directly affects the diversity and abundance of detected metabolites. According to Creydt et al. [7], extraction methods should be rapid, reproducible, and capable of extracting the widest possible range of metabolites while minimizing degradation to maximize the information obtained. Although various extraction techniques have been developed to obtain metabolite-rich extracts, particularly tannins, using different solvents and processing conditions, few studies have explored how different solvents influence the extraction and modulation of a diverse range of metabolites. For example, Chavan et al. [8] investigated the extraction of proanthocyanidins from beach pea using different solvents and found that acetone:water (7:3) mixture acidified with 1% hydrochloric acid maximized the extraction yield. Mailoa et al. [9], on the other hand, demonstrated that ethanol:water (3:7) was more effective than acetone-based mixtures for extracting proanthocyanidins from guava leaves. These results highlight the absence of a universal protocol for proanthocyanidin extraction. In addition, most existing methods focus specifically on phenolic compounds, particularly proanthocyanidins, without addressing the full range of metabolites present in *Acacia mearnsii* De Wild. bark. The choice of extraction solvent is also a critical factor, as it can directly influence the biological activities of plant extracts. For instance, Yagi et al. [10] investigated different solvents for metabolite extraction from *Phlomis* species and demonstrated that methanol was the most effective for recovering bioactive compounds, highlighting the importance of solvent selection for biological activity. Similarly, Llorent-Martínez et al. [11] reported that methanol was better than aqueous in enhancing antioxidant capacity and enzyme inhibition in eight *Salvia* species.

Given this context, the present study applied untargeted metabolomics with various solvents to explore the metabolite diversity of *Acacia mearnsii* De Wild. (black wattle) extracts. This research seeks to provide a broader understanding of its complex chemical composition, as well as to characterize the proanthocyanidin profile of black wattle bark cultivated in southern Brazil. By shedding light on the full range of metabolites, this study offers new insights that could expand the potential applications of *Acacia mearnsii* De Wild. in diverse industries, such as food, pharmaceuticals, and materials science, where its bioactive compounds may play an important role.

## Materials and methods

### Reagents and standards

Standards of gallic acid, ellagic acid, vanillic acid, caffeic acid, ferulic acid, 5-caffeoylquinic acid, *p*-coumaric acid, (+)-catechin, (+)-gallocatechin, (−)-epicatechin, (−)-epigallocatechin, (+)-gallocatechin-3-gallate, quercetin, naringenin, apigenin, resveratrol, daidzein, hydroxybenzoic acid, 2,5-dihydroxybenzoic acid, 3,5-dihydroxybenzoic acid, pyrogallol, kaempferol, protocathecuic acid, sinapic acid, trans-3-hydroxycinnamic acid, rutin hydrate, theogallin, phlorizin, fisetin, liquiritigenin, luteolin, myricetin, procyanidin A2, B1 procyanidin, B2 procyanidin, C1 procyanidin, citric acid, malic acid, quinic acid, ascorbic acid, and quercetin dihydrate were purchased from Sigma-Aldrich (purity > 90%) (St. Louis, MO). Acetonitrile with HPLC grade was from ScharLAB SL (Barcelona, Spain). Folin-Ciocalteu and vanillin were from Sigma-Aldrich (St. Louis, MO). Acetone, ethanol, and formic acid were obtained from Neon Comercial (São Paulo, Brazil). Methanol was acquired from Êxodo Científica (São Paulo, Brazil). Chloridric acid was purchased from Química Moderna (São Paulo, Brazil). Ultrapure water was generated by the Milipore System (Molsheim, FR).

### Samples

Black wattle bark samples (approximately 1 kg) were kindly supplied by TANAC (Montenegro, RS, Brazil), a company that produces natural extracts rich in tannins from renewable sources. The bark was sourced from *Acacia mearnsii* De Wild. trees grown in wattle forests in the State of Rio Grande do Sul, Brazil (32° 02′ 06″ S, 52° 05′ 55″ W).

To stabilize the fresh samples, the bark was frozen in an ultra freezer (Coldlab, CL200) and subsequently freeze-dried (L101, Liobras, Brazil). The dried samples were ground into a fine powder using a knife mill, vacuum sealed in bags (*Sulpack,* SVC 200 G2 model, Caxias do Sul, Brazil), and stored at −18 °C. The fine powder was then used for the extractions with different solvents.

### Solvent screening

To extract metabolites for the untargeted metabolomics study, eight different solvents were employed: distilled water at room temperature (25 °C, AW), distilled water heated to 85–90 °C (HW), and a series of binary mixtures—acetone:water (50:50 v/v, AC5), ethanol:water (50:50 v/v, E5), methanol:water (50:50 v/v, M5), acetone:water (70:30 v/v, AC7), ethanol:water (70:30 v/v, E7), and methanol:water (70:30 v/v, M7). All solvents were acidified with 0.35% formic acid. These solvents and/or mixtures were selected based on previous studies [8, 9]. A legend of sample names is available in Supplementary Material 1, Table S1.

### Untargeted metabolomics

#### Metabolites extraction

For metabolite extraction, 0.1 g of freeze-dried sample was combined with 1.5 mL of solvent in Eppendorf tubes. Prior to solvent addition, a small spatula of 0.5 mm glass beads was added to each sample. The mixture was agitated for 2 min at the maximum speed (150 G) using a Bead Ruptor™ 4 cell disruptor (OMNI International, USA). After agitation, the tubes were centrifuged at 13,416 g for 5 min (Eppendorf AG, Hamburg, Germany). The supernatants were carefully collected into new Eppendorf tubes. The solvents were removed from the extracts using a sample concentrator (New Lab NL-45-02, Piracicaba, São Paulo, Brazil) set to 40 °C for 45 min. The remaining aqueous extracts were resuspended in equal masses of distilled water and methanol to a total of 1000 mg (500 mg of water and 500 mg of methanol). The extracts were then filtered into amber vials*.*

The extraction procedure was carried out five times (five independent experiments). Pooled quality control (QC) samples were generated by combining 150 µL of each individual extract. Solvent and process blanks were included to account for any potential contamination from laboratory consumables. The solvent blank consisted of methanol:water (1:1) mixture, while process blanks were prepared by following the same procedure used for sample extraction, with distilled water substituted in place of the samples.

#### Data acquisition

Metabolomic experiments were carried out using an ultra-high performance liquid chromatography system (UHPLC, Nexera X2 Shimadzu, Kyoto, Japan), connected to an Impact II mass spectrometer (MS) (Impact II Bruker, Bruker Corporation, Massachusetts, EUA) equipped with a quadrupole-time-of-flight (QTOF) analyzer. An electrospray (ESI) was employed as an ionization source. Samples were randomized for the run order and analyzed both in the negative and positive electrospray modes.

The separation in the UHPLC was performed with an Acquity UPLC column HSS T3 (50 × 2.1 mm, 1.8 µm particle size), at the flow rate of 0.5 mL min^−1^ and column temperature of 40 °C. The analysis was performed with an injection volume of 5 μL per sample. The mobile phase consisted of solvents A (water acidified with 0.1% formic acid) and B (acetonitrile acidified with 0.1% formic acid). The gradient of mobile phases was the following: 0% B held for 0.5 min, a linear increase to 100% B over 8 min, 100% B held for 1 min, followed by a return to 0% B for 1 additional min. The injection volume of samples was 5 µL. Quality control (QC) samples were repeatedly injected to stabilize the instrument until consistent base peak chromatograms were achieved. Additionally, QC samples were analyzed every five sample injections (approximately once per hour) to assess intra-run variability.

The QTOF-MS parameters were set as follows: the scan range from *m/z* 50 to 1000 Da, drying gas temperature of 200 °C, nebulizer gas pressure of 4.5 bar, drying gas flow at 10 L.min^−1^. MS/MS spectra were acquired in data-dependent acquisition (DDA) mode with a threshold of 1500. Instrument control and raw data processing were carried out using Esquire Control and Data Analysis 4.3 software, respectively (Bruker Daltonics, Billerica, MA, USA). Raw data were calibrated internally using sodium formate clusters. MSMS were acquired using the same MS settings, employing auto MS/MS scanning mode with a collision energy of 10 eV.

#### Data pre-processing

The files obtained from the data of UHPLC-QTOF-MS analysis were originally obtained in *.d format. These data were then converted to *.mzML format using Data Analysis 4.3 software (Bruker Daltonics, Billerica, MA, USA). The data in *.mzML format was processed using MZmine 4.5.20 software [12] for peak deconvolution. The process includes the stages of mass detection, ADAP (automated data analysis pipeline) chromatogram building, chromatogram deconvolution, isotope grouping, alignment of chromatograms, gap filling, and removing duplicate features. All parameters are described in Supplementary Material 1, Tables S2 and S3. After the data had been processed, the file obtained (.csv) was exported for data analysis.

#### Data analysis

Data filtering was performed in Excel software (2501 version). Features that did not exhibit at least a tenfold increase in quality control (QC) samples compared to process blanks were excluded from the analysis. Subsequently, features with a coefficient of variation greater than 30% in the QCs were also removed. The final feature table containing MS1 data for both negative and positive ionization modes is provided in Tables S4 and S5 (Supplementary Material 1).

Data analysis was carried out using MetaboAnalyst 6.0 software (https://www.metaboanalyst.ca/) [13]. Features were normalized based on the mass of the resuspended extracts (approximately 1 g). A log (base 10) transformation was then applied to the data. Both univariate and multivariate statistical analyses were performed using MetaboAnalyst 6.0 software, including principal component analysis (PCA), heatmap generation, and volcano plots.

#### Metabolite annotation

Metabolite annotation was performed using the MS/MS spectra data, following the Metabolomics Standard Initiative guidelines. In this process, three approaches were used: manual annotation, library searches, and in silico annotation. For the manual approach, the following parameters were considered: elution order in the T3 column, accurate mass, and fragmentation pattern compared to the standards and/or data available in literature. In this case, Compass DataAnalysis 4.3 software was used. Additionally, data in the DataAnalysis software was converted to *.mzML format and uploaded to the Global Natural Products Social Network (GNPS) platform (https://gnps2.org) [14], where the algorithm Library Search was employed. To generate the molecular network, a mass tolerance of 2.0 Da for precursor ions was used. Ion fragment mass variances were set to 0.5 Da for clustering each group of acquired MS/MS spectra. Nodes were connected only if the cosine score exceeded 0.7, with a minimum of 6 matching peaks in the fragmentation spectrum. The molecular network spectra were then compared to the GNPS spectral libraries, where the same data parameters were applied to the sample spectra. Furthermore, Sirius software (version 5.8.6) was used, and the function Canopus Class Prediction was enabled.

#### Dataset availability

The raw MS data have been deposited in MetaboLights under the access number MTBLS13040 [15], while the MS/MS data are available in GNPS MassIVE repository with the number MSV000098533 [14].

### Phenolic compounds quantification and characterization

#### Total phenolic compounds

Total phenolic compounds (TPC) were assessed according to the methodology developed by Singleton and Rossi [16] with modifications. The method was modified in terms of the total volume of samples and reaction time.

The extracts were prepared following the procedure described in "Metabolites extraction section”. After the extraction, 0.25 mL of 2 N Folin-Ciocalteu reagent was added to 0.25 mL of each sample, along with 1.5 mL of ultrapure water. This mixture was allowed to react for 5 min before the addition of 1 mL of 7% sodium carbonate solution. The mixture was stored for 2 h at room temperature and in the dark before measuring the absorption at 765 nm using a UV–visible spectrophotometer (Biospectro SP-220 – Eikonal do Brasil, São Paulo, Brazil). Gallic acid was used as a standard, and total phenolic content was expressed as milligram equivalents of gallic acid per gram of sample (mg GAE g^−1^). All samples were analyzed in triplicate.

#### Total proanthocyanidin content

The total content of proanthocyanidins (TPAC) was measured using the vanillin method, following the procedure discussed by Price et al. [17], with some modifications. The extracts were prepared following the procedure described in “Metabolites extraction.” After extraction, 0.4 mL of each extract was mixed with 0.6 mL of methanol and 3 mL of vanillin working reagent. This reagent was prepared on the day of the analysis by mixing equal volumes of an 8% HCl solution in methanol and a 1% vanillin solution in methanol. After that, mixtures were kept for 10 min at 30 °C in a water bath, protected from light. Absorbances were measured at 490 nm using a UV–visible spectrophotometer (Biospectro SP-220 – Eikonal do Brasil, São Paulo, Brazil). Catechin was used as a standard, and total condensed tannin content was expressed as milligram equivalents of catechin per gram of sample (mg CE g^−1^). All samples were analyzed in triplicate.

#### Proanthocyanidin annotation

The presence of high-molecular-weight proanthocyanidin oligomers was evaluated by an LC–MS methodology. For extraction, 0.1 g of ground samples was mixed with 1.5 mL of methanol:water (8:2, v/v) acidified with 0.35% formic acid. The obtained extract was analyzed in an HPLC (Shimadzu, Kyoto, Japan) equipped with a diode-array detector (DAD, Shimadzu, SPD-20^a^) and a quadrupole-time-of-flight (QTOF) mass spectrometer with an electrospray ionization source (ESI – Bruker Daltonics, micrOTOF-QIII, Bremen, Germany). Separation was carried out with a Phenomenex C18 column (250 mm × 4.6 mm, 4 µm). Mobile phases were classified as A (ultrapure water acidified with 0.1% formic acid) and B (acetonitrile acidified with 0.1% formic acid). The extract was eluted according to a binary gradient, starting at 99:1 (v/v) of A/B, followed by 50:50 (v/v) of A/B over 50 min, and ending with 1:99 (v/v) of A/B for 5 min. The total run time was 55 min, with a flow rate of 0.7 mL min^−1^ at 29 °C and an injection volume of 20 µL.

Mass spectra were acquired in scan mode over an *m/z* range of 50 to 1500. The MS acquisition parameters were the following: ESI source operating in the negative ionization mode, capillary voltage of 3500 V, dry gas temperature (N_2_) at 310 °C with a flow rate of 8 L min^−1^, and nebulizer gas pressure of 4 bar.

Annotation of proanthocyanidins was performed using the GNPS platform (see Metabolite annotation section)  and manual interpretation, based on accurate mass, MSMS fragmentation patterns, elution order, and UV–vis absorption characteristics.

#### Statistical analysis

Comparisons of the data were performed through ANOVA followed by Tukey’s test, with a 95% significance level (*Statistica,* 13.0 version, Statsoft Inc., Tulsa, USA).

## Results and discussion

### Impact of different solvents on metabolite extraction

After data pre-processing and filtering, 3106 and 4284 chemical features were retained for analysis in the negative and positive ionization modes, respectively. Initially, 4756 features were detected in the negative mode and 23,910 in the positive mode. Thus, 65.3% of features were retained in the negative mode and 17.9% in the positive mode after filtering. Figures S1a and S1b (Supplementary Material 2) display the principal component analysis (PCA) of all samples and features, including QCs (Quality Control samples), in the negative and positive ionization modes, respectively. These figures demonstrate the suitability of the input data. The overlap of the pooled QCs (light green color) in both ionization modes suggests good performance of the mass spectrometer and consistency in the data cleaning process, indicating reliable measurements across identical samples. Replicates from the different treatments (solvents) demonstrated lower reproducibility compared to QC samples, which is expected as they represent independent biological replicates. Moreover, the complexity of the extraction process, which involves multiple steps, including sample concentration and re-suspension, likely contributes to the increased variance between samples.

Figures S2a and S2b (Supplementary Material 2) show the boxplots of log10-transformed relative feature abundance data for negative and positive modes, respectively, with samples grouped by treatment type. These figures corroborate the aforementioned behavior, demonstrating consistent measurement of identical QCs.

**Fig. 1:**
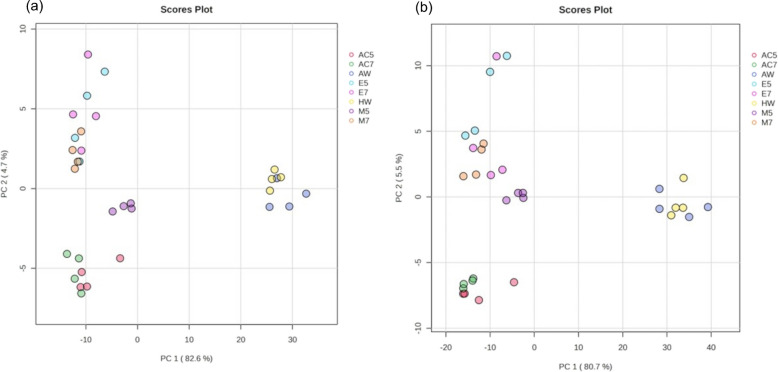
Principal components analysis (PCA) score plots obtained for the different extraction solvents: **a** score plot in negative ionization mode and **b** score plot in positive ionization mode. Extraction solvents: ambient water (AW, water at room temperature), hot water (HW, water at 90 °C), 50% acetone (AC5), 70% acetone (AC7), 50% ethanol (E5), 70% ethanol (E7), 50% methanol (M5), and 70% methanol (M7)

The PCA scores plot without QC samples for all samples and features can be found in Fig. 1 for MS data in negative (Fig. 1a) and positive (Fig. 1b) ionization modes. As can be seen, the PCA score plots exhibit similar behavior between the negative and positive ionization modes. The formation of two distinct clusters in both ionization modes demonstrates differences in the relative abundances of chemical features when water was used as a solvent compared to when organic extraction solvents were employed. Although metabolites can ionize differently depending on the ionization mode, the analysis will primarily focus on the negative mode, with occasional references to the positive mode, as similar trends were observed in both.

As shown in Fig. 1a, principal component 1 (PC1) accounts for 82.6% of the total variance, while principal component 2 (PC2) explains 4.7%, showing that dimensionality reduction from thousands to 2 allows explanation of approximately 87% of the variance between groups. As mentioned above, the plot presents a clear separation along PC1 between aqueous solvents and organic solvents, forming two different clusters. In addition, Fig. 1a shows a separation between ethanolic solvents and acetone or methanol-based ones (except 70% methanol). This pattern suggests that the distinct solvents are modulating the extraction of metabolites from black wattle bark in different ways, showing differences when water or organic solvents were used.

**Fig. 2:**
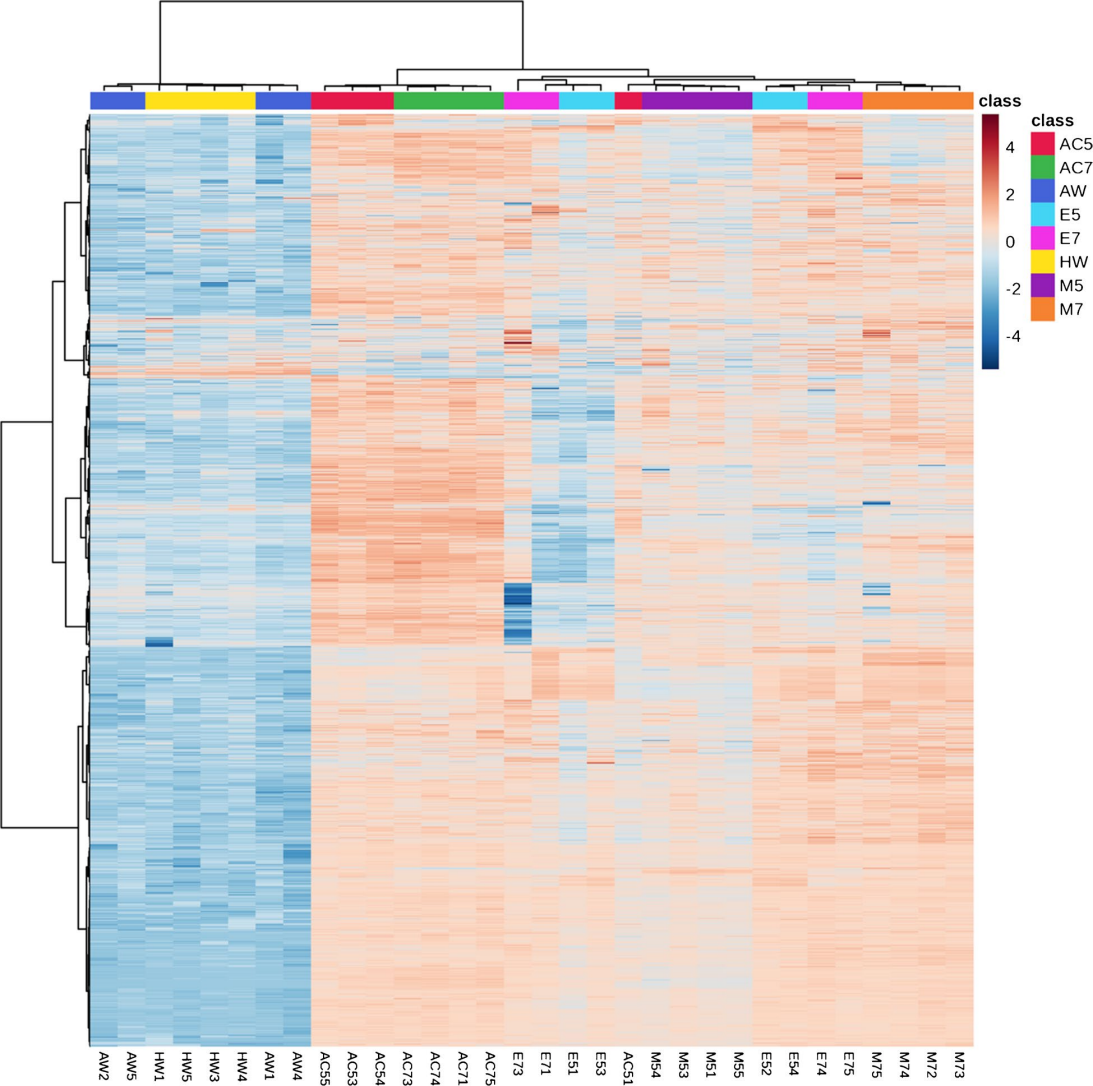
Heatmap of all significantly different features at an FDR-corrected *p*-value ≤ 0.05 detected by untargeted metabolomics using LC–MS in negative ionization mode. Hierarchical clustering using Euclidean distances and Ward’s linkage method was used horizontally to categorize samples and vertically to group features

Figure 2 presents a heatmap of all significantly different features at a false discovery rate (FDR) corrected *p*-value ≤ 0.05 in negative mode, clustering both samples (columns) and features (rows). This figure shows that a visual summary of the data highlights distinct patterns in compound relative abundance across different solvents applied. The first vertical cluster divides the heatmap into two distinct regions based on the treatments (solvents). The first region, on the left side, includes water solvents in two conditions: at room temperature (AW) and heated to 90 °C (HW). This zone predominantly exhibits a blue color, indicating a lower relative abundance of the extracted features.

In contrast, the middle and right sections of the heatmap form the second cluster, characterized by predominantly red hues, suggesting that organic solvents yielded higher relative feature abundances. Notably, an even more intense red zone is observed in the middle, corresponding to extractions performed with acetone at both 50% and 70% concentrations. This suggests that acetone was more effective in extracting certain features compared to the other solvents.

Table 1 shows eighty (80) features that were annotated as phenolic compounds, organic acids, and lignans, among other compounds, accounting for 2.6% of the features detected in negative ionization mode and 1.8% of those detected in positive ionization mode. The metabolite profile of black wattle bark reveals a diverse distribution across multiple classes. These bioactive compounds include both primary and secondary metabolites. Regarding primary metabolites, carbohydrates, amino acids, and fatty acid derivatives were annotated. Among secondary metabolites, organic acids, including gallic, malic, protocatechuic, and citric acids, were annotated. Flavonoids such as (+)-catechin, (+)-gallocatechin, (+)-robinetinidol, and fisetinidol, along with proanthocyanidins in dimeric and trimeric forms, highlight the presence of condensed tannins in the bark. Additionally, coumarins, such as esculetin, as well as other phenolic derivatives, like vanillin, contribute to the metabolic complexity of this matrix.

**Table 1:** Annotated metabolites in *Acacia mearnsii* De Wild. bark, including parameters such as retention time, molecular formula, experimental ions (positive and negative modes), beyond experimental errors, fragmentation patterns, and annotation levels

ID	Compound	*t*_***R***_ (min)^a^	Molecular formula	Positive mode			Negative mode			Level^e^
				Experimental [M + H]^+b^	Error (ppm)	MS/MS (*m/z*)	Experimental [M − H]^− c^	Error (ppm)	MS/MS (*m/*z)^d^	
1	Pipecolic acid	0.3	C_6_H_11_NO_2_	130.0864	−1.1	84.0807 (100%) 56.0488 (52.39%)	n.d.^f^	n.c.^g^	n.d	2
2	Hydroxypipecolic acid	0.3	C_6_H_11_NO_3_	146.0814	−1.8	82.0652 (100%) 55.0541 (46.77%) 67.0428 (38.28%) 100.0754 (18.48%)	144.0670	−2.6	n.d	3
3	Valine	0.3	C_5_H_11_NO_2_	118.0864	−0.9	58.0647 (100%) 70.0650 (15.33%)	n.d	n.c	n.d	3
4	Malic acid	0.3	C_4_H_6_O_5_	n.d	n.c	n.d	133.0146	−2.7	115.0037 (100%) 133.0157 (55.58%) 71.0141 (21.49%) 89.0231 (8.07%)	2
5	Sucrose + formic acid	0.4	C_13_H_24_O_13_	n.d	n.c	n.d	387.1148 [M + HCOO]^−^	−1.0	341.1088 (100%) 179.0585 (25.30%) 119.0361 (4.46%)	2
6	Glucoronic acid + formic acid	0.4	C_8_H_16_O_8_	n.d	n.c	n.d	239.0775 [M + HCOO]^−^	−1.2	193.0735 (100%) 89.0246 (58.17%)	3
7	Citric acid	0.6	C_6_H_8_O_7_	n.d	n.c	n.d	191.0201	−1.8	111.0084 (100%) 87.0079 (66.44%) 191.0203 (56.68%)	2
8	Flavonoid C-hexoside	0.6	C_20_H_20_O_10_	421.1143	−1.4	n.d	419.0982	0.4	299.0564 (100%) 311.0526 (57.60%) 329.0654 (49.27%) 419.0970 (35.99%)	3
9	Hydroxybenzoic acid derivative	1.0	C_8_H_8_O_5_	n.d	n.c	n.d	183.0294	2.5	183.0296 (100%) 139.0383 (98.98%)	2
10	Gallic acid	1.2	C_7_H_6_O_5_	n.d	n.c	n.d	169.0144	−1.1	125.0248 (100%) 169.0143 (41.79%) 79.0191 (4.45%)	1
11	Benzoic acid derivative	1.4	C_8_H_6_O_5_	n.d	n.c	n.d	181.0145	−1.3	137.0241 (100%) 109.0302 (61.46%) 135.0072 (48.24%) 183.0296 (27.12%)	3
12	Methyl citrate	1.6	C_7_H_10_O_7_	n.d	n.c	n.d	205.0356	−1.3	111.0097 (100%) 161.0037 (83.28%) 162.9821 (54.49%) 130.9974 (45.82%) 143.0362 (44.89%) 83.0122 (28.48%)	2
13	Protocathecuic acid	2.1	C_7_H_6_O_4_	n.d	n.c	n.d	153.0195	−1.0	153.0194 (100%)	1
14	Galloyl hexoside	2.2	C_13_H_16_O_10_	n.d	n.c	n.d	331.0671	−0.2	271.0462 (100%) 331.0674 (90.76%) 211.0248 (51.03%) 153.0196 (43.29%) 169.0152 (36.73%) 125.0245 (12.58%)	2
15	Syringic acid hexoside	2.3	C_15_H_20_O_10_	383.0955 (M + Na)	−0.6	140.0471 (100%) 155.0711 (48.08%) 125.0233 (34.56%)	359.0985	−4.5	197.0458 (100%) 182.0231 (28.03%) 138.0333 (25.16%) 153.0553 (16.12%) 123.0085 (7.16%) 167.0002 (5.66%)	2
16	(+)-Gallocatechin	2.3	C_15_H_14_O_7_	307.0820	−2.4	n.d	305.0670	1.0	125.0247 (54.17%) 179.0352 (44.16%) 137.0245 (30.22%) 139.0401 (27.37%)	1
17	Gentisic acid hexoside	2.3	C_13_H_16_O_9_	n.d	n.c	n.d	315.0723	−0.4	152.0116 (46.58%) 153.0182 (20.67%) 108.0214 (13.20%)	2
18	Flavonoid-O-hexoside	2.3	C_18_H_26_O_12_	n.d	n.c	n.d	433.1353	−0.3	289.0717 (100%) 329.0680 (51.46%) 433.1348 (42.25%) 301.0931 (33.27%)	3
19	(−)-Epigallocatechin	2.4	C_15_H_14_O_7_	307.0818	−1.9	n.d	305.0672	−1.8	n.d	1
20	Catechin hexoside	2.4	C_21_H_24_O_11_	n.d	n.c	n.d	451.1252	−1.3	n.d	3
21	Proanthocyanidin dimer (1)	2.4	C_30_H_26_O_13_	595.1473	−4.6	139.0392 (100%) 271.0609 (52.21%) 241.0502 (34.71%) 163.0392 (26.46%)	593.1302	−0.2	305.0666 (100%) 177.0194 (61.71%) 593.1291 (46.61%) 425.0870 (22.96%)	2b
22	Alpha amino acid derivative	2.4	C_16_H_19_N_5_O_6_	378.1401	1.8	140.0469 (100%) 181.0499 (65.18%) 85.0286 (30.56%)	n.d	n.c	n.d	3
23	Vanillin	2.5	C_8_H_8_O_3_	153.0552	−3.5	63.0229 (100%) 110.0361 (61.21%)	n.d	n.c	n.d	2
24	(+)-Catechin	2.5	C_15_H_14_O_6_	291.0871	−2.5	139.0331 (100%) 123.0397 (41.13%)	289.0720	−0.7	123.0474 (100%) 109.0322 (67.92%) 151.0430 (33.86%) 137.0256 (33.33%)	1
25	Hesperetin dihydrochalcone hexoside	2.5	C_22_H_26_O_11_	467.1565	−0.5	n.d	465.1405	−0.5	303.0874 (100%) 166.0271 (21.24%) 138.0333 (16.34%) 288.0642 (6.50%)	3
26	Dihydrophaseic acid derivative	2.5	C_21_H_32_O_10_	n.d	n.c	n.d	443.1925	−0.6	443.1920 (100%) 444.1955 (21.88%)	3
27	(Epi)catechin hexoside	2.6	C_21_H_24_O_11_	453.1404	−2.7	n.d	451.1245	0.2	289.0717 (100%) 401.1456 (28.45%) 139.0406 (28.30%) 451.1249 (20.78%)	3
28	(+)-Robinetinidol	2.6	C_15_H_14_O_6_	n.d	n.c	n.d	289.0719	−0.3	109.0323 (100%) 139.0410 (79.71%) 125.0265 (52.52%) 149.0250 (25.05%)	2b
29	Proanthocyanidin dimer (2)	2.6	C_30_H_26_O_12_	579.1523	−4.4	123.0443 (100%) 139.0381 (91.19%) 271.0609 (81.55%) 241.0477 (65.71%) 229.0508 (30.24%) 393.0983 (18.68%)	577.1351	0.1	289.0721 (100%) 177.0197 (76.16%) 577.1344 (40.61%) 245.0830 (34.14%) 409.0928 (14.10%)	2b
30	(−)-Epicatechin	2.7	C_15_H_14_O_6_	291.0864	−0.2	n.d	289.0723	−1.7	n.d	1
31	Proanthocyanidin dimer (3)	2.7	C_30_H_26_O_11_	563.1567	−3.5	123.0441 (100%) 135.0484 (36.62%) 241.0496 (36.54%) 139.0381 (35.76%) 393.0981 (11.93%)	561.1403	−0.1	289.0719 (100%) 561.1407 (50.72%) 409.0928 (31.60%) 245.0830 (22.78%) 161.0247 (17.51%)	2b
32	Proanthocyanidin trimer (1)	2.7	C_45_H_38_O_19_	883.2153	−8.3	n.d	881.1934	0.1	305.0668 (100%) 881.1920 (97.12%) 177.0194 (50.87%) 407.0775 (45.63%) 713.1502 (22.69%) 593.1289 (19.88%)	2b
33	Fisetinidol	2.8	C_15_H_14_O_5_	n.d	n.c	n.d	273.0770	−0.7	123.0452 (100%) 149.0231 (67.04%) 273.0782 (40.59%)	3
34	Hydroxycoumarin	2.8	C_9_H_6_O_3_	163.0395	−3.1	91.0544 (100%) 107.0492 (66.46%) 65.0390 (57.47%) 117.0576 (12.32%)	n.d	n.c	n.d	3
35	Hesperetin dihydrochalcone	2.8	C_16_H_16_O_6_	305.1025	−0.5	n.d	303.0877	−0.9	166.0274 (100%) 138.0336 (73.15%) 288.0646 (21.44%)	3
36	Proanthocyanidin trimer (2)	2.8	C_45_H_38_O_18_	867.2219	−10.2	n.d	865.1983	0.2	865.1974 (100%) 289.0718 (52.95%) 577.1343 (35.41%) 177.0195 (27.21%) 529.1136 (21.82%) 391.0820 (20.26%)	2b
37	Proanthocyanidin trimer (3)	3.0	C_45_H_38_O_17_	851.2270	−10.4	n.d	849.2028	0.9	289.0720 (100%) 849.2035 (91.15%) 391.0827 (30.71%) 529.1142 (30.36%) 177.0196 (29.79%) 577.1352 (19.42%)	2b
38	(−)-Epirobinetinidol	3.1	C_15_H_14_O_6_	n.d	n.c	n.d	289.0715	0.8	109.0317 (100%) 139.0413 (74.61%) 125.0249 (55.41%) 149.0249 (25.49%)	2b
39	Proanthocyanidin trimer (4)	3.1	C_45_H_38_O_16_	835.2337	−12.5	n.d	833.2077	1.2	289.0719 (100%) 833.2080 (72.16%) 391.0830 (34.09%) 561.1397 (28.49%) 681.1602 (24.06%) 161.0243 (21.64%)	2b
40	Eriodicytol O-hexoside + formic acid	3.1	C_23_H_28_O_12_	n.d	n.c	n.d	495.1507 [M + HCOO]^−^	0.2	287.0927 (100%) 183.0333 (9.74%)	2
41	Isokaempferide	3.3	C_16_H_12_O_6_	301.0712	−0.2	n.d	299.0553	−1.8	284.0310 (100%) 255.0288 (36.86%) 227.0330 (20.51%) 299.0565 (17.03%)	3
42	Fisetin	3.3	C_15_H_10_O_6_	287.0552	−0.8	n.d	285.0403	0.7	285.0393 (100%) 135.0084 (24.40%) 259.0591 (20.50%) 121.0315 (14.38%)	1
43	Diacetylphloroglucinol hexoside	3.3	C_17_H_24_O_9_	373.1501	−2.0	n.d	371.1348	−0.1	209.0822 (100%) 165.0921 (14.23%)	2
44	Phlorizin	3.3	C_21_H_24_O_10_	n.d	n.c	n.d	435.1294	0.3	273.0771 (100%) 167.0353 (36.37%)	1
45	Phlorisovalerophenone	3.3	C_11_H_14_O_4_	211.0967	−1.2	68.9973 (100%) 123.0442 (75.98%) 163.0393 (38.43%) 139.0399 (36.32%) 155.0342 (19.99%)	209.0823	−1.5	n.d	3
46	Diacetylphloroglucinol hexoside + formic acid	3.3	C_18_H_26_O_11_	n.d	n.c	n.d	417.1406 [M + HCOO]^−^	−0.8	209.0823 (100%) 371.1349 (19.68%)	2
47	Phloretin	3.4	C_15_H_14_O_5_	275.0915	−0.5	n.d	273.0770	0.7	167.0352 (100%) 273.0768 (14.90%)	2
48	Pharboside C	3.5	C_27_H_44_O_11_	n.d	n.c	n.d	543.2800	2.0	335.2229 (100%) 543.2812 (48.66%) 289.0 728(7.92%)	3
49	Isorhamnetin	3.6	C_16_H_12_O_7_	n.d	n.c	n.d	315.0507	1.2	300.0266 (100%) 255.0296 (52.98%) 298.0471 (46.03%) 315.0512 (29.06%)	3
50	Quercetin	3.6	C_15_H_10_O_7_	n.d	n.c	n.d	301.0352	0.4	299.0560 (100%) 284.0333 (72.09%) 301.0327 (48.16%) 285.0770 (39.43%) 151.0022 (36.40%)	1
51	Liquiritigenin	3.6	C_15_H_12_O_4_	257.0815	−0.7	n.d	255.0661	0.2	119.0509 (100%) 135.0083 (93.10%) 255.0667 (75.48%) 120.0553 (14.02%)	1
52	Abscisic acid	3.6	C_15_H_20_O_4_	n.d	n.c	n.d	263.1289	0.1	219.1380 (100%) 220.1423 (79.50%) 201.1274 (60.00%) 149.0604 (58.00%) 153.0904 (34.50%)	2
53	Galangin	3.6	C_15_H_10_O_5_	271.0604	−1.1	137.0238 (100%) 65.0387 (76.09%) 271.0605 (45.63%) 197.0593 (33.52%) 149.0230 (24.13%) 215.0715 (19.98%) 225.0527 (11.95%)	269.0457	−0.4	269.0458 (100%) 270.0483 (17.15%) 136.0489 (4.78%)	2
54	Terpene glycoside	3.7	C_32_H_36_O_15_	n.d	n.c	n.d	659.1982	−0.1	311.0925 (100%) 209.0818 (49.75%) 177.0198 (48.14%) 497.1454 (41.67%) 659.1957 (19.05%)	3
55	Flavonoid derivative	3.7	C_16_H_10_O_8_	331.0453	−1.3	n.d	329.0292	3.3	314.0072 (100%) 270.9888 (88.19%) 298.9824 (59.76%) 329.0308 (42.61%) 327.2211 (37.80%)	2
56	Butin	3.8	C_15_H_12_O_5_	273.0762	−0.5	137.0234 (100%) 81.0337 (57.09%) 107.0462 (41.90%) 89.0377 (40.72%)	271.0610	0.6	135.0451 (100%) 271.0612 (23.21%)	2
57	Naringenin	3.9	C_15_H_12_O_5_	273.0761	−0.3	153.0183 (100%) 91.0546 (32.89%) 147.0438 (15.98%) 271.0587 (7.63%)	271.0611	0.6	271.0601 (100%) 151.0034 (76.95%) 119.0506 (22.01%)	1
58	Kaempferol	3.9	C_15_H_10_O_6_	n.d	n.c	n.d	285.0400	1.6	285.0407 (100%)	1
59	Medium-chain Hydroxy acid derivative	3.9	C_11_H_20_O_5_	n.d	n.c	n.d	231.1238	0.2	231.1253 (100%) 233.0867 (67.72%) 141.1285 (55.09%) 87.0073 (48.42%)	3
60	Isoliquiritigenin	4.1	C_15_H_12_O_4_	257.0812	−0.4	107.0490 (100%) 91.0543 (43.40%) 137.0234 (35.20%)	255.0659	0.3	151.0402 (100%) 135.0089 (76.15%) 255.0653 (60.60%) 119.0515 (49.49%)	2
61	Davidigenin	4.1	C_15_H_14_O_4_	259.0963	0.8	n.d	257.0814	2.1	151.0396 (100%) 257.0804 (8.58%) 135.0089 (3.10%) 107.0487 (2.44%)	3
62	Fatty Acid Derivative	4.1	C_18_H_34_O_5_	n.d	n.c	n.d	329.2327	2.1	329.2325 (100%) 171.1020 (22.49%)	2
63	Aminoalcohol	4.3	C_20_H_43_NO_4_	362.3276	−3.1	300.2893 (100%) 70.0651 (75.30%) 132.1014 (68.97%) 256.2634 (64.35%) 146.1187 (55.27%)	n.d	n.c	n.d	3
64	Phloretin + formic acid	4.4	C_16_H_14_O_7_	n.d	n.c	n.d	317.0667 [M + HCOO]^−^	1.0	167.0341 (100%) 315.1604 (25.84%)	2
65	Fatty acyl	4.8	C_17_H_26_O_4_	n.d	n.c	n.d	293.1755	1.1	221.1534 (100%) 220.1463 (62.13%) 236.1060 (56.62%)	2
66	Diterpenoid	4.9	C_20_H_30_O_5_	n.d	n.c	n.d	349.2015	1.6	349.1991 (100%) 287.1998 (44.59%) 351.2195 (44.65%)	3
67	Diterpenoid	4.9	C_20_H_26_O_2_	299.2012	−2.3	145.1011 (100%) 91.0541 (23.19%)	n.d	n.c	n.d	3
68	Diterpenoid	5.1	C_20_H_30_O_4_	335.2220	−0.8	89.0598 (100%) 201.1642 (73.91%) 145.1004 (62.14%) 119.0850 (61.05%)	333.2063	2.5	333.2062 (100%) 334.2070 (20.83%) 245.1545 (3.40%) 315.1940 (3.20%)	2
69	Lignan derivative	5.1	C_24_H_30_O_6_	415.2122	−1.8	119.0856 (100%) 135.0793 (8.70%)	n.d	n.c	n.d	2
70	Emodin	5.2	C_15_H_10_O_5_	n.d	n.c	n.d	269.0447	3.3	269.0454 (100%) 270.0508 (16.07%) 225.0559 (12.36%)	2
71	Hexadecanedioic acid	5.5	C_16_H_30_O_4_	n.d	n.c	n.d	285.2064	2.7	285.2071 (100%) 267.1976 (75.72%) 223.2052 (35.33%)	2
72	Oxooctadecadienoic acid derivative	5.6	C_18_H_30_O_3_	n.d	n.c	n.d	293.2117	1.9	293.2117 (100%) 275.2002 (69.77%) 235.1698 (42.02%) 157.0133 (28.63%) 183.1362 (28.37%) 121.1043 (5.85%)	2
73	Coriolic acid	5.9	C_18_H_32_O_3_	n.d	n.c	n.d	295.2267	3.9	295.2269 (100%) 277.2199 (41.15%) 183.0111 (17.06%) 195.1381 (10.57%)	2
74	Fatty acyl glycoside	6.2	C_27_H_46_O_10_	n.d	n.c	n.d	529.3003	2.8	529.3004 (100%) 279.2319 (87.24%)	3
75	Esculetin	6.5	C_9_H_6_O_4_	179.0343	−2.4	n.d	177.0187	3.5	177.0189 (100%) 133.0290 (80.06%) 130.9665 (39.98%) 105.0334 (28.46%)	2
76	Atranorin	6.6	C_19_H_18_O_8_	n.d	n.c	n.d	373.0920	0.9	177 (100%) 163 (44.13%) 195 (12.64%)	2
77	Hydroxy acid derivative	6.9	C_15_H_22_O_4_	n.d	n.c	n.d	265.1470	−9.5	265.1469 (100%) 96.9585 (7.66%)	2
78	Coumarin derivative	7.0	C_19_H_36_O_4_	n.d	n.c	n.d	327.2529	3.5	327.2531 (100%) 325.1828 (83.61%) 283.2633 (60.68%) 183.0113 (33.29%)	2
79	Linolenic acid	7.1	C_18_H_30_O_2_	279.2324	−1.8	n.d	277.2166	2.4	277.2170 (100%) 279.1626 (57.55%) 278.2167 (22.31%)	2
80	Long-chain fatty acid	7.4	C_22_H_42_O_4_	n.d	n.c	n.d	369.2998	3.5	369.2999 (100%) 307.2992 (31.27%) 351.2904 (20.49%)	3

^a^Retention time on the Acquity UPLC T3 column and solvent gradient of 0.1% formic acid in water and acetonitrile with 0.1% formic acid

^b^Annotated metabolites in the positive ionization mode

^c^Annotated metabolites in the negative ionization mode

^d^Fragmentation pattern MSMS

^e^Identification confidence level according to Schymanski et al. [27]

^f^n.d., not detected

^g^n.c., not calculated

This diverse composition underscores the multifunctional nature of black wattle bark, particularly its potential antioxidant and bioactive properties. While many studies have primarily focused on its proanthocyanidin content, Table 1 reveals a significantly more complex and diverse chemical profile than previously reported. A more detailed discussion of black wattle bark characterization is provided in “Quantification and characterization of phenolic compounds found in black wattle bark.”

**Fig. 3:**
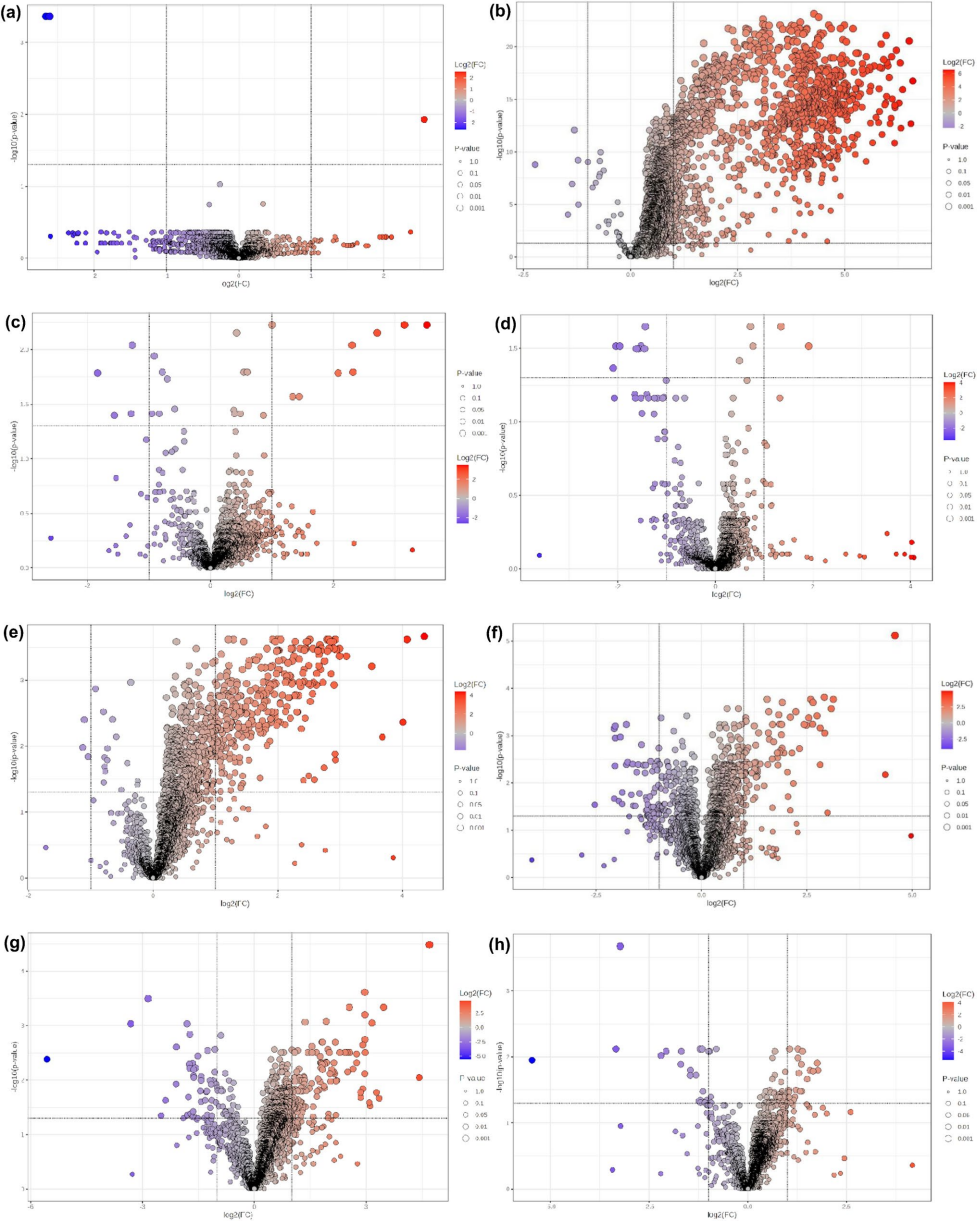
Volcano plot of features detected by untargeted metabolomics analysis in negative ionization mode comparing: **a** water at room temperature (AW) versus hot water (HW); **b** aqueous versus organic solvents; **c** 50% acetone (AC5) versus 70% acetone (AC7); **d** 50% ethanol (E5) and 70% ethanol (E7); **e** 50% methanol (M5) versus 70% methanol (M7); **f** 70% acetone (AC7) versus 70% methanol (M7); **g** 70% acetone (AC7) versus 50% ethanol (E5); and **h** 70% methanol (M7) versus 50% ethanol (E5)

For a feature-wise analysis, the results of univariate *t*-tests comparing treatments (solvents) are visually represented as volcano plots, which display the *p*-values from significance testing combined with relative fold changes in abundance (Fig. 3). The horizontal and vertical dotted lines represent the cutoff values for multiple testing corrected adjusted *p*-value of 0.05 and fold change of 1, respectively. These figures take both the magnitude of change and variability into consideration [18]. These analyses were conducted to elucidate solvent-induced differences in terms of the number of features, considering the effects of temperature, solvent concentration, and solvent type.

**Fig. 4:**
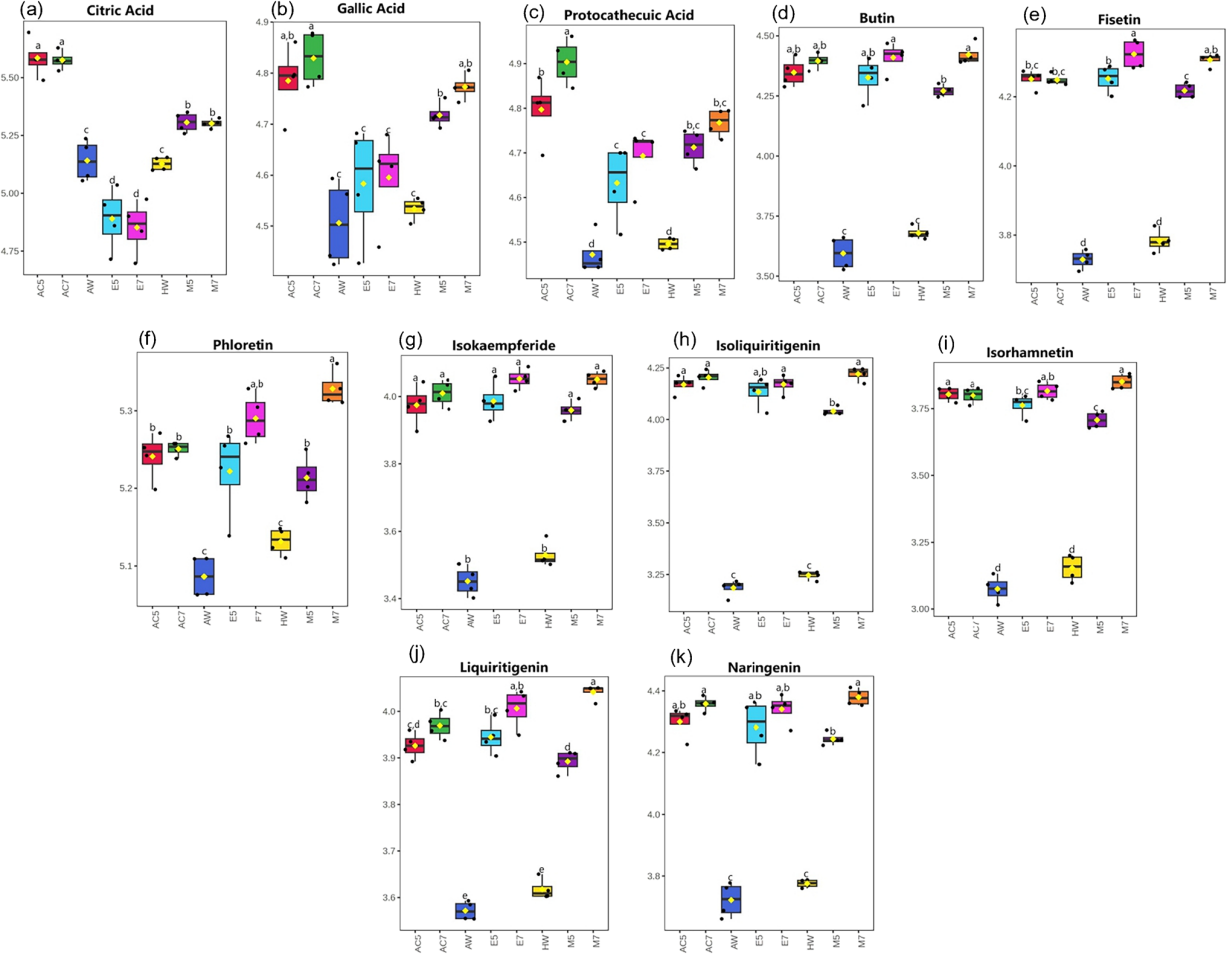
Boxplots comparing the relative abundance of organic acids and flavonoids among treatments (ANOVA and Tukey’s honest significant difference tests, *α* = 0.05). Lines represent the median abundance per treatment. **a** Citric acid; **b** gallic acid; **c** protocathecuic acid; **d** butin; **e** fisetin; **f** phloretin; **g** isokampferide; **h** isoliquiritigenin; **i** isorhamnetin; **j** liquiritigenin; **k** naringenin

**Fig. 5:**
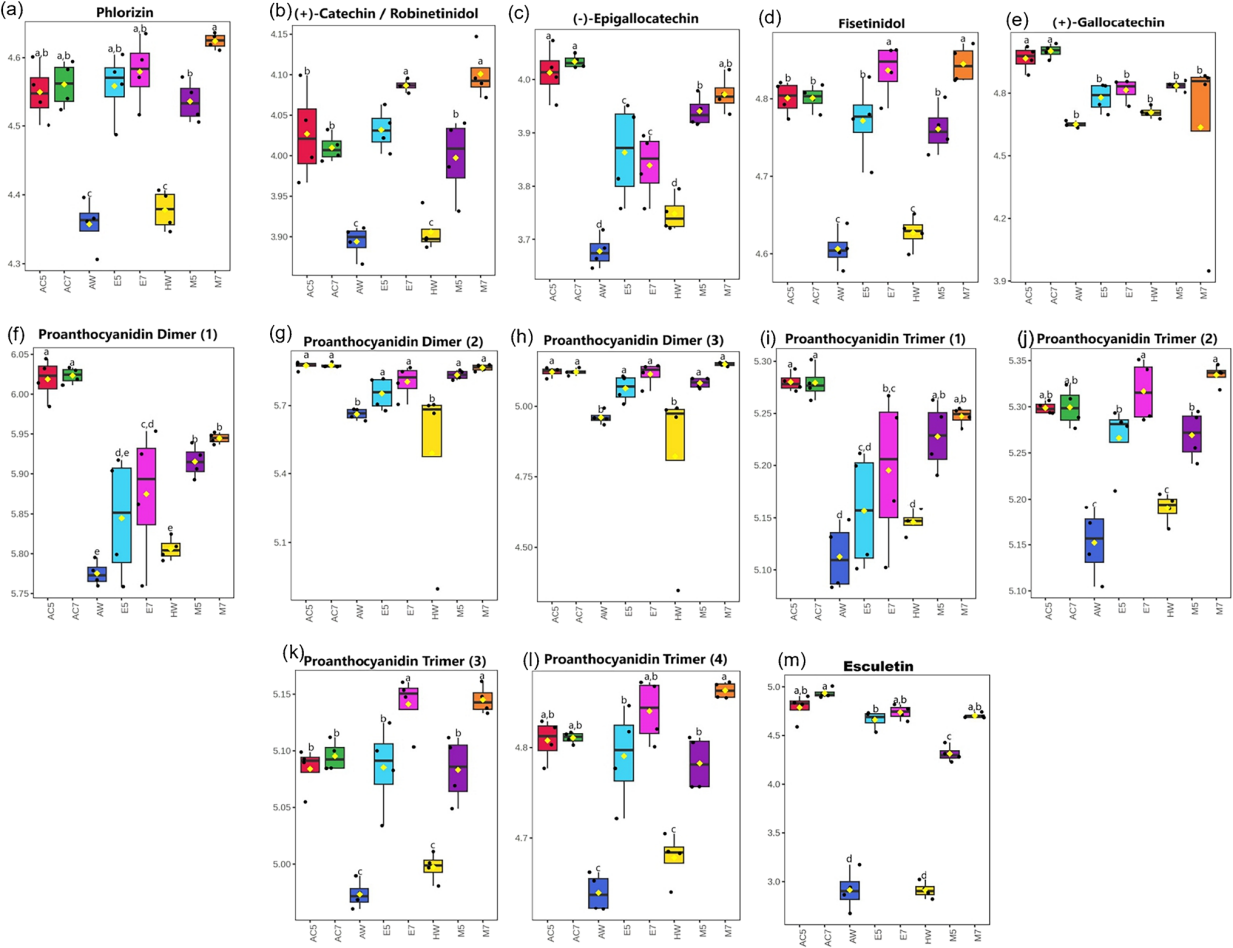
Boxplots comparing the relative abundance of phenolic glycosides (**a**), monomers of proanthocyanidins (**b**, **c**, **d**, and **e**), dimers of proanthocyanidins (**f**, **g**, and **h**), trimers of proanthocyanidins (**i**, **j**, **k**, and **l**), and other metabolites (**m**) among treatments (ANOVA and Tukey’s honest significant difference tests, *α* = 0.05). Lines represent the median abundance per treatment. **a** Phlorizin; **b** catechin/robinetinidol; **c** epigallocatechin; **d** fisetinidol; **e** gallocatechin; **f** proanthocyanidin dimer 1; **g** proanthocyanidin dimer 2; **h** proanthocyanidin dimer 3; **i** proanthocyanidin trimer 1; **j** proanthocyanidin trimer 2; **k** proanthocyanidin trimer 3; **l** proanthocyanidin trimer 4; **m** esculetin

When comparing room temperature water (AW) to hot water (HW) (Fig. 3a), only three features were significantly different, demonstrating that heating did not affect the extraction of compounds. In contrast, Fig. 3b reveals that 1205 features significantly differed between aqueous and organic solvents, corroborating the differences previously discussed. This trend is also evident when analyzing the behavior of some specific compounds. Figures 4 and 5 present boxplots of the relative abundance of selected compounds across all treatments. Some of these compounds exhibited statistically significant differences among treatments, as determined by ANOVA (*α* = 0.05) followed by Tukey’s test. Particularly, gallic acid and protocatechuic acid showed lower relative abundances when extracted with ambient-temperature or hot water (Fig. 4). Interestingly, 50% and 70% ethanol were the least effective solvents for citric acid extraction. Figure 4 also displays boxplots of the relative abundance of flavonoids (the major group identified in *Acacia mearnsii* De Wild. bark in this study) across all treatments. Once again, room temperature and hot water were the least effective solvents. This pattern was also observed for phenolic glucoside compounds and proanthocyanidin monomers (Fig. 5), proanthocyanidin dimers and trimers (Fig. 5), and other metabolites such as esculetin (Fig. 5).

The results obtained for water, both at room temperature and under heating, can be explained by two opposing effects: (1) increased extraction efficiency due to temperature and (2) thermal degradation of heat-sensitive metabolites. The balance between these effects ultimately resulted in no significant difference in overall metabolite yield. According to Snyder’s theory, solvent properties, particularly polarity and dielectric constant, play a critical role in solute solubility [19]. Increasing the temperature generally enhances extraction efficiency by reducing solvent viscosity, allowing better penetration into the plant matrix and increasing solute diffusivity, thereby facilitating the migration of metabolites into the solvent phase. Moreover, heating water slightly reduces its polarity, which may favor the extraction of moderately polar compounds. Ibañez et al. [20] reported that high temperatures can decrease the dielectric constant of water to values similar to those of methanol or acetonitrile, thereby increasing the solubility of less polar compounds.

Conversely, Snyder’s framework also suggests that temperature can influence solute stability. Many plant metabolites, especially some phenolic compounds, are thermolabile and may undergo oxidation, transformation, hydrolysis of conjugated forms, or polymerization at elevated temperatures [21]. At the temperature range applied in this study (85–90 °C), thermal effects on phenolic compounds are already detectable, as supported by the Folin–Ciocalteu results discussed in “Quantification and characterization of phenolic compounds found in black wattle bark,” which indicate heat-induced losses in total phenolic content. Similar behavior has been reported in the literature, where phenolic compounds exhibit temperature-dependent degradation, often following first-order kinetics, affecting both their concentration and antioxidant activity [22, 23]. Together, these opposing mechanisms, improved extractability and concurrent thermal degradation, explain the comparable metabolite yields observed between room temperature and heated water extractions.

The superior performance of organic solvents compared to water in metabolite extraction can also be explained using Snyder’s solvent theory [19]. According to the author, solvents are classified based on their polarity index, which influences their ability to dissolve specific types of compounds. Water is a highly polar solvent, making it effective for extracting hydrophilic compounds, such as organic acids. However, many bioactive metabolites present in *Acacia mearnsii* De Wild. bark, such as flavonoids and tannins, have intermediate to low polarity. Organic solvents (like acetone, ethanol, and methanol), when used in aqueous mixtures (e.g., 50% or 70% solutions), provide a balanced polarity that enhances the solubility of both polar and moderately nonpolar metabolites. This polarity modulation allows the extraction of a broader range of compounds, including those that are not effectively solubilized in pure water. In addition to solubility effects, organic solvents can alter the physical structure of the plant matrix, improving metabolite release by disruption of hydrogen bonds and cell wall components, and improve penetration into plant tissues. This effect is particularly relevant for proanthocyanidins and flavonoids, which are often bound to structural components of the plant matrix.

For organic solvents, when comparing different concentrations of the same solvent, only 13 features significantly differed between 50 and 70% acetone (AC5 and AC7), with nine being more abundant in 70% acetone (Fig. 3c). This suggests that concentration changes had a minimal impact on extraction. A similar trend was observed in Fig. 3d, where only ten features were significantly different between 50 and 70% ethanol (E5 and E7), with eight showing higher relative abundance in 50% ethanol. However, Fig. 3e highlights a different pattern for methanol: 306 features were significantly different between 50 and 70% methanol (M5 and M7), indicating that methanol concentration influenced compound extraction. Particularly, 70% methanol resulted in 303 features with higher relative abundance than 50% methanol. Overall, Fig. 3a–e demonstrates that the most pronounced differences in extracted features occur when comparing water-based extractions to organic solvents.

To further compare the differences between organic solvents, we focused on those that exhibited the highest number of significantly upregulated features when relative abundance was considered (Fig. 3f–h). Figure 3f shows that the comparison between 70% acetone (AC7) and 70% methanol (M7) resulted in 135 significantly different features, with 82 exhibiting higher relative abundance in 70% acetone than in 70% methanol. In contrast, Fig. 3g reveals that comparing 70% acetone (AC7) to 50% ethanol (E5) increased the number of significantly different features to 166; in this case, 123 features showed higher abundance in 70% acetone when compared to 50% ethanol. Moreover, Fig. 3h indicates that only 39 features present significant differences between 70% methanol (M7) and 50% ethanol (E5), 21 showing higher relative abundance in 70% methanol, suggesting that both solvents had a similar influence on compound extraction.

Overall, 70% acetone (AC7) extracted the highest number of features with elevated relative abundances, highlighting its effectiveness as an extraction solvent. This result agrees with those reported by Chavan et al. [8], who described that 70% acetone was the best solvent to extract the highest concentration of bioactive compounds from beach pea. This trend may be explained by the polar and aprotic nature of acetone when compared to other solvents. According to Mokrani and Madani [24], acetone is a more efficient solvent to extract compounds with a high molecular weight, such as condensed tannins. Furthermore, Tourabi et al. [25] described that acetone is highly effective for extracting compounds from plant material, as it can solubilize molecules across a broad polarity range. However, as demonstrated by Meneses et al. [26], acetone:water mixtures are more efficient than pure acetone for extracting certain compounds, such as phenolics. This increased efficiency is attributed to the higher polarity of the mixture, which creates a more favorable medium for solubilizing polar compounds, as previously discussed. Nevertheless, water remains widely used as an extraction solvent for metabolites such as condensed tannins in industrial applications, owing to its low cost and environmental sustainability. When considering the use of acetone:water mixtures on a food or feed industrial scale, it is crucial to evaluate strategies for acetone removal and subsequent purification of the bioactive compounds of interest.

Figure 4 shows that 70% acetone yielded the highest extraction efficiency for all organic acids. For (+)-gallocatechin and (−)-epigallocatechin (Fig. 5), both 50% and 70% acetone were the most suitable solvents for extraction. Similarly, 50% and 70% acetone maximized the relative abundance of proanthocyanidin dimers (Fig. 5), coumarins, among others (Fig. 5). However, for flavonoids (Fig. 4), 70% acetone was not the most effective solvent for certain compounds, such as fisetin, phloretin, and liquiritigenin. Instead, 70% methanol (M7) and 70% ethanol (E7) appeared to be the most effective solvents when considering the full range of flavonoids. For (+)-catechin, (+)robinetinidol, and fisetinidol, 70% ethanol and 70% methanol were also the most efficient extraction solvents (Fig. 5).

For phlorizin, a phloretin derivative, 50% and 70% acetone, as well as 70% methanol, were the most effective extraction solvents. Additionally, for phlorizin, 50% and 70% ethanol also proved to be viable alternatives (Fig. 5). For proanthocyanidin trimer, in almost all cases, 50% and 70% acetone, along with 70% ethanol and 70% methanol, were suitable solvents for trimer extraction (Fig. 5).

These findings highlight how different solvents influence compound extraction in distinct ways and are consistent with the patterns observed in the heatmap analysis (Fig. 2). Creydt et al. [7] also demonstrated that there is not a single protocol when the objective is the extraction of polar and nonpolar metabolites. Overall, organic solvents are more effective in extracting metabolites from black wattle bark. Among them, 50% and 70% acetone, along with 70% methanol, yielded the highest metabolite feature abundances.

### Quantification and characterization of phenolic compounds found in black wattle bark

**Fig. 6:**
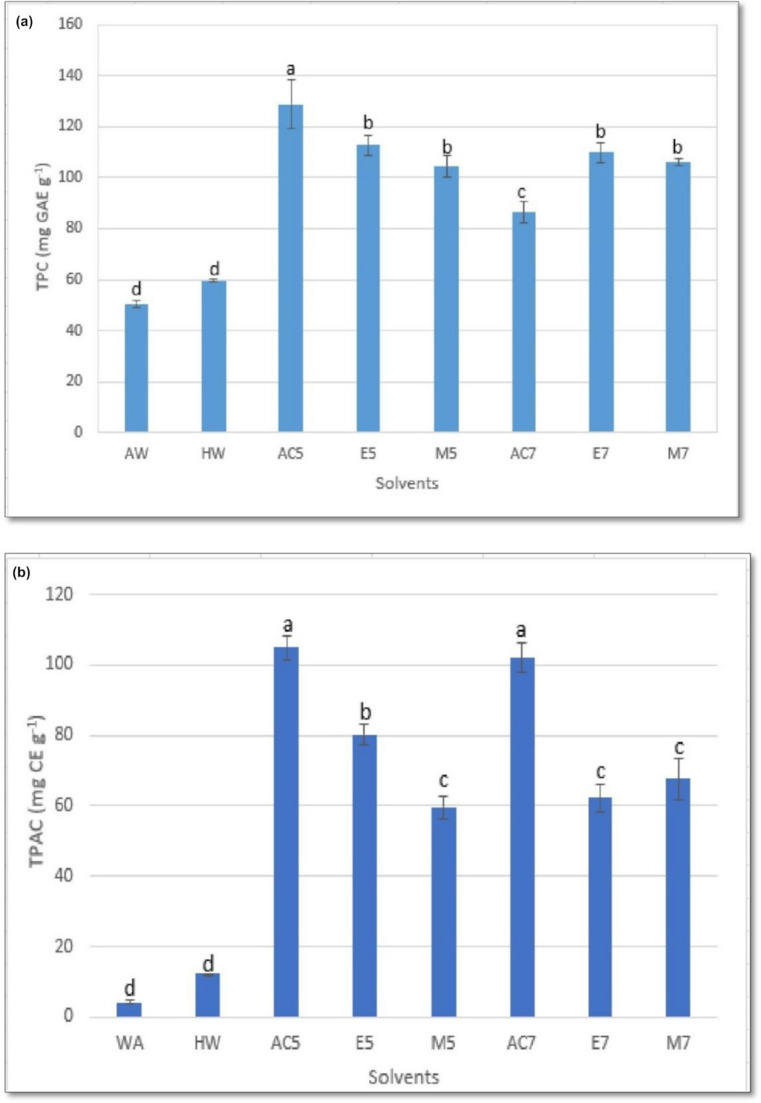
Total phenolic compounds (**a**) and total proanthocyanidins (**b**) in black wattle bark using different extraction solvents. *Different letters in the same plot represent significant differences (*p* < 0.05) between data

In addition to the untargeted metabolomic analysis, total phenolic compounds (TPC) and total proanthocyanidin content (TPAC) analyses were performed using the previously investigated extraction solvents to characterize black wattle bark and assess solvent-dependent differences. Figure 6a and b present the results for TPC and TPAC, respectively.

The choice of the extraction solvent significantly influenced TPC and TPAC contents (*p* < 0.05). Among them, 50% acetone (AC5) yielded the highest TPC value, while TPAC was highest when using acetone at both 50% and 70% concentrations (AC5 and AC7, respectively). However, it is noteworthy that 70% acetone (AC7) resulted in the lowest TPC among the organic solvents. In line with this, Bhebhe et al. [28] reported that 50% acetone promoted higher extraction of total phenolic compounds than absolute acetone when analyzing black tea and selected infusions. According to the authors, this trend may be attributed to the solubility of natural phenols in solvents with intermediate polarity. In contrast, water, whether at room temperature (AW) or heated up to 90 °C (HW), produced the lowest values of both TPC and TPAC. Ethanol and methanol, at both concentrations, showed no statistically significant differences in TPC and TPAC values.

Although these results were expected, they hold great relevance, particularly considering that water is commonly used for proanthocyanidin extraction on an industrial scale due to its environmentally friendly nature. However, it is important to highlight that 50% acetone (AC5) demonstrated the highest efficiency in extracting phenolic compounds and proanthocyanidins, which could have important economic implications for optimizing extraction processes.

Several phenolic compounds identified in black wattle bark were annotated for the first time in this study for this raw material (Table 1), including naringenin, kaempferol, and liquiritigenin. This highlights the richness of its extracts in bioactive compounds. These compounds are well known for their strong antioxidant properties and other biological activities, such as antimicrobial, anti-obesity, and anti-diabetic effects [29]. Other flavonoids, such as quercetin, have been previously reported in this matrix [30]. Additionally, compounds like isokaempferide, phloretin, and phlorizin were also identified in this study (Table 1). Butin, a flavonoid isomer of naringenin, was detected as well, confirming earlier findings by Roux and Paulus [31] in wattle heartwoods. Furthermore, Kusano et al. [6] identified several low-molecular-weight constituents in *Acacia mearnsii* De Wild. bark, including syringic acid and butin, being the last one also detected here, while syringic acid was verified here linked to a hexoside. To our knowledge, no recent studies have reported additional phenolic compounds in black wattle bark extracts beyond proanthocyanidins. The diversity of phenolics found here emphasizes the potential of this matrix as a natural source of bioactive compounds of interest to the food and feed industries, not only to enhance antioxidant and biological properties in product development, but also to contribute technological functionalities. In animal nutrition, recent studies have explored the inclusion of condensed tannins as feed additives due to their antimicrobial properties and potential as growth promoters [4]. Although these biological activities are typically attributed to condensed tannins, the findings of this study reveal that black wattle bark contains numerous other bioactive compounds beyond proanthocyanidins, which may also contribute to these effects. This underscores the importance of further exploring and discussing these newly identified compounds.

Proanthocyanidin biosynthesis is part of the flavonoid pathway, which originates from the phenylpropanoid metabolic route [32, 33]. The process begins with the deamination of phenylalanine, leading to the formation of naringenin chalcone, which establishes the characteristic C3-C6-C3 skeleton of flavonoids [32, 34]. A series of enzymes, including hydrolases, synthases, and isomerases, regulate this pathway, generating intermediate and final metabolites such as naringenin, apigenin, quercetin, and kaempferol. These compounds then give rise to leucoanthocyanidins, the precursors of flavan-3-ol monomers (e.g., catechin, gallocatechin, robinetinidol, and fisetinidol). Through polymerization and interflavanic interactions, these monomers form proanthocyanidins [32, 34].

This biosynthetic pathway explains the presence of various flavonoids as intermediates or final products in black wattle bark. The identification of naringenin, kaempferol, and liquiritigenin, reported here for the first time in this matrix, further supports the complex and diverse nature of its secondary metabolite composition.

Regarding proanthocyanidins, four monomeric units were identified in the present study: catechin, gallocatechin, robinetinidol, and fisetinidol. These findings align with previous reports [3, 35, 36]. It is well established that catechin and gallocatechin serve as the starter units of proanthocyanidins in black wattle bark, while robinetinidol and fisetinidol function as extender units [35, 36].

In this study, epicatechin and epigallocatechin were annotated for the first time in this raw material which is consistent with the genus as these monomers are commonly found in plant barks. We hypothesize that these diastereoisomers could act as additional starter units in proanthocyanidin biosynthesis. The presence of these diastereoisomers was unexpected, as Roux [37] previously reported their absence in black wattle bark. To the best of our knowledge, no recent studies have confirmed the presence of (−)-epicatechin and (−)-epigallocatechin in this matrix.

**Fig. 7:**
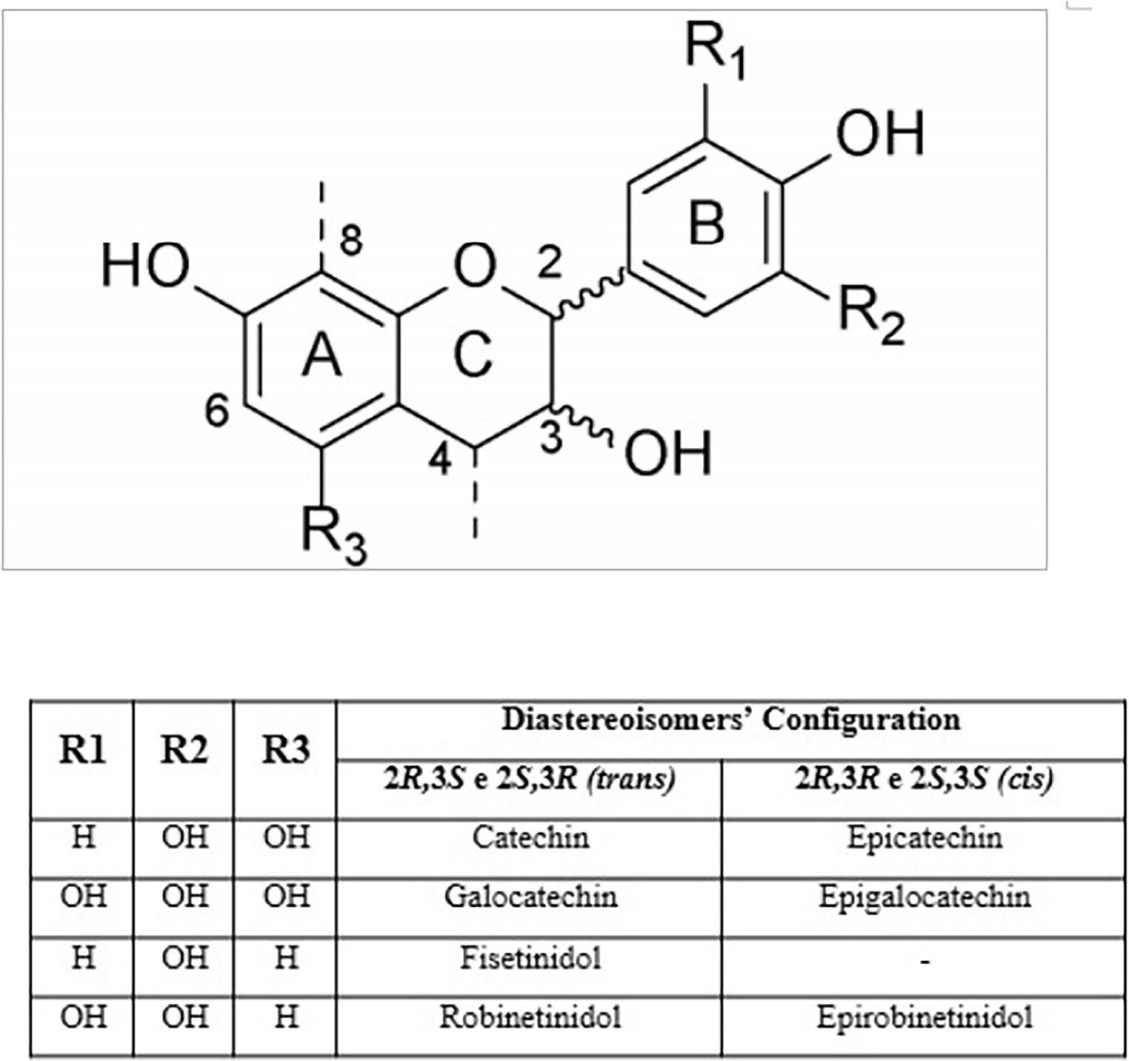
Flavan-3-ols monomers found in black wattle bark

The molecular structures of the flavan-3-ol monomers identified in black wattle bark in this study are presented in Fig. 7.

The application of MS in both negative and positive ionization modes enabled the detection of oligomers up to trimers in black wattle bark. Pasch et al. [38] employed MALDI-TOF/MS to analyze condensed tannin extracts from various sources. They detected oligomers up to octamers for *Acacia mearnsii* De Wild. bark. Given the expectation of larger oligomers, an additional analysis was conducted in the present study, using LC-DAD-MS and a C18 column. This approach confirmed the annotation of monomers, dimers, and trimers, while also revealing the presence of tetramers and pentamers. Table 2 presents the estimated compositions of the oligomers identified in black wattle bark, based on MS data previously reported in the literature [3].

**Table 2:** Proposed composition of oligomers obtained from black wattle bark*

Oligomer	Starter unit	Starter unit	Extender unit	Extender unit	Adduct [M−H]^−^
	(Epi)catechin	(Epi)gallocatechin	(Epi)robinetinidol	Fisetinidol	
Dimers	1	0	0	1	561
	1	0	1	0	577
	0	1	0	1	577
	0	1	1	0	593
Trimers	1	0	0	2	833
	1	0	1	1	849
	1	0	2	0	865
	0	1	1	1	865
	0	1	2	0	881
Tetramers	1	0	3	0	1153
	0	1	2	1	1153
	0	1	3	0	1169
	1	0	3	1	1425
	0	1	2	2	1425
Pentamers	1	0	4	0	1441
	0	1	3	2	1441
	0	1	4	0	1457

*The chemical composition of proanthocyanidins was proposed according to MS characteristics and from what was described by Venter et al. [3]

Three dimer peaks were observed at *m/z* 561, 577, and 593 in the MS spectra of black wattle bark extracts under negative ionization mode. For trimers, four oligomers were detected at *m/z* 833, 849, 865, and 881. Regarding tetramers, two oligomers were identified at *m/z* 1153 and 1169. These results differ from those reported by Venter et al. [3], who identified five potential tetramers in *Acacia mearnsii* De Wild. bark. Such compositional differences may be attributed to variations in sample origin and geographic conditions, as Venter et al. [3] analyzed South African extracts, whereas this study focused on Brazilian *Acacia mearnsii* De Wild.

Cornering pentamers, three compounds were detected, appearing as clusters separated by 16 Da, a pattern also observed for dimers, trimers, and tetramers. The pentamers were detected at *m/z* 1425, 1441, and 1457. As described by Venter et al. [3], the chemical composition of proanthocyanidins from black wattle bark is more complex than that of other sources, such as quebracho, which consists of a catechin starter unit and a fisetinidol extender unit. These structural differences suggest that proanthocyanidins from different raw materials may have distinct applications in the food and feed industries.

Monomers, including (epi)catechin and (epi)gallocatechin, were annotated by comparison with authentic standards, while (epi)robinetinidol and fisetinidol were annotated by diagnostic and by comparison with literature data. In this context, fisetinidol exhibited a characteristic fragment peak at *m/z* 149 (negative mode), as reported by Abilkassymova et al. [39].

Regarding the annotation of (epi)catechin and (epi)robinetinidol, it is necessary to analyze certain characteristics in greater depth. In this context, Supplementary Material 2 presents figures illustrating the practices adopted for annotating these compounds in the bark of the black wattle. In Fig. S3, for the annotation of (+)-catechin (Peak 1) and (−)-epicatechin (Peak 3), analytical standards available in our laboratory were employed, enabling level 1 identification. Key features, including retention time, accurate mass, and MSMS fragmentation patterns, were consistent between the standards and the corresponding metabolites in the sample. Figure S3 presents the extracted ion chromatogram (EIC) at *m/z* 289 obtained using the LC–MS method described in "Data acquisition" (method A—Fig. S3a) and “Proanthocyanidin annotation” (method B—Fig. S3b). Figure S3c shows the overlay of chromatograms from the sample and the analytical standards obtained by method B, highlighting the similarity in retention times between the two diastereoisomers and their corresponding standards. For the annotation of (+)-robinetinidol (peak 2) and (−)-epirobinetinidol (peak 4), no analytical standards were available; therefore, the assignment was based on their MSMS fragmentation patterns, which differ from those of (+)-catechin (peak 1) and (−)-epicatechin (peak 3). It is important to note that we assumed that the fragmentation patterns of (+)-robinetinidol and its diastereoisomer, (−)-epirobinetinidol, were the same, similarly to what we assumed for (+)-catechin (peak 1) and (−)-epicatechin (peak 3). Therefore, when referring to fragmentation patterns, the notation (±)-(epi)catechin or (±)-(epi)robinetinidol will be used. In addition, it was assumed that the elution order of the two diastereoisomers followed the same behavior observed for (+)-catechin (peak 1) and (−)-epicatechin (peak 3). Method B applied a high collision energy (35 eV), which allowed the precursor ions of *m/z* 289.0720 (negative mode) to undergo more intense fragmentation. Figure S4 shows the results obtained in MSMS for (+)-catechin (Fig. S4a) and (+)-robinetinidol (Fig. S4b). As can be seen from these figures, (+)-catechin and (+)-robinetinidol showed different fragmentation patterns. While (+)-catechin has the most intense MSMS fragments at *m/z* 109, 123, and 137, (+)-robinetinidol has the most intense MSMS fragments at *m/z* 109, 125, and 139. Thus, although the metabolites mentioned have the same precursor ion (289.0720 Da in negative mode), the differences in fragmentation patterns show that the R groups (hydroxyls, for example) are attached to different locations in the molecules. Furthermore, it is important to mention that both compounds underwent RDA (Retro-Diels-Alder) fragmentation, from which rings A and B are released from the molecules. Figure S5 and Fig. S6 show the fragmentation pathways mentioned for these molecules, respectively.

These described monomers form the structural foundation of the annotated dimers, trimers, tetramers, and pentamers. As shown in Table 2, the oligomers in black wattle bark are predominantly prorobinetinidins (robinetinidol), consisting of a catechin or gallocatechin starter unit and a robinetinidol extender unit. In the present study, the diastereoisomers epicatechin and epigallocatechin were also identified, suggesting their potential role as additional starter units. Furthermore, oligomers containing fisetinidol as an extender unit were also annotated. According to Venter et al. [3], in dimers, the extender units are linked to the more reactive C-8 position of the starter unit, while in trimers, the additional extender unit may also bind to the remaining C-6 position.

The results of the present study corroborate these findings while also highlighting that *Acacia mearnsii* De Wild. bark extracts contain a diverse range of bioactive compounds beyond proanthocyanidins. These insights could serve as a valuable tool in the development of food and feed products with enhanced antioxidant properties.

## Conclusion

This study explored the metabolic composition of *Acacia mearnsii* De Wild. (black wattle) bark, uncovering a phytochemical complexity that extends beyond proanthocyanidins. The choice of extraction solvent played a crucial role in metabolite recovery, with 70% acetone proving to be the most effective in extracting a diverse range of compounds. These results emphasize the importance of optimized extraction protocols in metabolomic studies.

A total of 80 metabolites were identified, including flavonoids, organic acids, and other bioactive compounds, with several being reported in black wattle bark for the first time. The characterization of proanthocyanidins confirmed the presence of monomers, (epi)catechin and (epi)gallocatechin as starter units, as well as dimers, trimers, tetramers, and pentamers, with (epi)robinetinidol and fisetinidol as extender units, demonstrating the structural diversity of condensed tannins in this material.

These findings highlight the potential of black wattle bark extracts for applications in the food and feed industries, particularly due to their abundance of bioactive compounds with antioxidant and other beneficial properties. Furthermore, the identification of previously unreported metabolites underscores the need for further research into the technological and functional potential of this raw material.

## Supplementary Information

Below is the link to the electronic supplementary material.

Supplementary file1 (XLSX 2.01 MB)

Supplementary file2 (DOCX 1.66 MB)

## Data Availability

The raw MS data have been deposited in MetaboLights under the access number MTBLS13040, while the MS/MS data are available in GNPS MassIVE repository with the number MSV000098533.

## Glossary

AC5: Acetone:water 50:50 v/v
AC7: Acetone:water 70:30 v/v
AW: Distilled water at room temperature (25 °C)
E5: Ethanol:water 50:50 v/v
E7: Ethanol:water 70:30 v/v
ESI: Electrospray ionization source
FDR: False discovery rate
HW: Distilled hot water (85–90 °C)
M5: Methanol:water 50:50 v/v
M7: Methanol:water 70:30 v/v
MS: Mass spectrometer
PCA: Principal component analysis
PC1: Principal component 1
PC2: Principal component 2
QC: Pooled quality control samples
QTOF: Quadrupole-time-of-flight analyzer
TPAC: Total proanthocyanidins content
TPC: Total phenolic compounds
UHPLC: Ultra-high performance liquid chromatography system

## References

[1] Griffin AR, Midgley SJ, Bush D, Cunningham PJ, Rinaudo AT. Global uses of Australian acacias – recent trends and future prospects. Divers Distrib. 2011;17:837–47. 10.1111/j.1472-4642.2011.00814.x.

[2] Chan JM, Day P, Feely J, Thompson R, Little KM, Norris CH. *Acacia mearnsii* industry overview: current status, key research and development issues. South For. 2015;77:19–30. 10.2989/20702620.2015.1006907.

[3] Venter PB, Senekal ND, Kemp G, Amra-Jordaan M, Khan P, Bonnet SL, et al. Analysis of commercial proanthocyanidins. Part 3: the chemical composition of wattle (*Acacia mearnsii*) bark extract. Phytochemistry. 2012;83:153–67. 10.1016/j.phytochem.2012.07.012. 22917955 10.1016/j.phytochem.2012.07.012

[4] Fraga-Corral M, Otero P, Echave J, Garcia-Oliveira P, Carpena M, Jarboui A, et al. By-products of agri-food industry as tannin-rich sources: a review of tannins’ biological activities and their potential for valorization. Foods. 2021;10(1):137. 10.3390/foods10010137. 33440730 10.3390/foods10010137 PMC7827785

[5] Molino S, Casanova NA, Henares JAR, Miyakawa MEF. Natural tannin wood extracts as a potential food ingredient in the food industry. J Agric Food Chem. 2020;68:2836–48. 10.1021/acs.jafc.9b00590. 31117489 10.1021/acs.jafc.9b00590

[6] Kusano R, Ogawa S, Matsuo Y, Tanaka T, Yazaki Y, Kouno I. α-amylase and lipase inhibitory activity and structural characterization of acacia bark proanthocyanidins. J Nat Prod. 2011;74:119–28. 10.1021/np100372t. 21192716 10.1021/np100372t

[7] Creydt M, Arndt M, Hudzik D, Fischer M. Plant metabolomics: evaluation of different extraction parameters for nontargeted UPLC-ESI-QTOF-Mass Spectrometry at the example of white *Asparagus officinalis*. J Agric Food Chem. 2018;66:12876–87. 10.1021/acs.jafc.8b06037. 30411896 10.1021/acs.jafc.8b06037

[8] Chavan UD, Shahidi F, Naczk M. Extraction of condensed tannins from beach pea (*Lathyrus maritimus* L.) as affected by different solvents. Food Chem. 2001;75:509–12. 10.1016/S0308-8146(01)00234-5.

[9] Mailoa MN, Mahendradatta M, Laga A, Djide N. Tannin extract of guava leaves (*Psidium guajava* L) variation with concentration organic solvents. IJSTR. 2013;2:106–10.

[10] Yagi S, Zengin G, Cetiz MV, Cziáky Z, Jeko J, Bahsi M, et al. Chemical characterization, antioxidant and enzyme-inhibitory activities of different extracts from three *Phlomis* species. Chem Open. 2025;14:e202500004. 10.1002/open.202500004. 10.1002/open.202500004 PMC12957883 40351016

[11] Llorent-Martínez EJ, Nilofar, Sinan KI, Darendelioglu E, Bahsi M, Polat R, Çakılcıoğlu U, Orlando G, Ferrante C, Zengin G. Incorporating the HPLC-ESI-Q-TOF-MS profiles with the biochemical properties of eight *Salvia* species. eFood. 2025;6:e70021. 10.1002/efd2.70021.

[12] Schmid R, Heucheroth S, Korf A, Smirnov A, Myers O, Dyrlund TS, et al. Integrative analysis of multimodal mass spectrometry data in MZmine 3. Nat Biotechnol. 2023;41:447–9. 10.1038/s41587-023-01690-2. 36859716 10.1038/s41587-023-01690-2 PMC10496610

[13] Chong J, Wishart DS, Xia J. Using metaboanalyst 4.0 for comprehensive and integrative metabolomics data analysis. Curr Protoc Bioinformatics. 2019. 10.1002/cpbi.86. 31756036 10.1002/cpbi.86

[14] Wang M, Carver JJ, Phelan VV, Sanchez LM, Garg N, Peng Y, et al. Sharing and community curation of mass spectrometry data with Global Natural Products Social Molecular Networking. Nat Biotechnol. 2016;34:828–37. 10.1038/nbt.3597. 27504778 10.1038/nbt.3597 PMC5321674

[15] Yurekten O, Payne T, Tejera N, Amaladoss FX, Martin C, Williams M, et al. Metabolights: open data repository for metabolomics. Nucleic Acids Res. 2024;52:D640-46. 10.1093/nar/gkad1045. 37971328 10.1093/nar/gkad1045 PMC10767962

[16] Singleton VL, Rossi JA. Colorimetry of total phenolics with phosphomolybdic-phosphotungstic acid reagents. Am J Enol Vitic. 1965;16:144–58. 10.5344/ajev.1965.16.3.144.

[17] Price ML, Van Scoyoc S, Butler LG. A critical evaluation of the vanillin reaction as an assay for tannin in sorghum grain. J Agric Food Chem. 1978;26:1214–8. 10.1021/jf60219a031.

[18] Whitney K, Gracia-Gonzalez G, Simsek S. Stability of wheat floret metabolites during untargeted metabolomics studies. Metabolites. 2022;12:62. 10.3390/metabo12010062. 35050184 10.3390/metabo12010062 PMC8780833

[19] Snyder LR, Carr PW, Rutan SC. Solvatochromically based solvent-selectivity triangle. J Chromatogr A. 1993;656:537–47. 10.1016/0021-9673(93)80818-S.

[20] Ibañez E, Kubátová A, Señoráns FJ, Cavero S, Reglero G, Hawthorne SB. Subcritical water extraction of antioxidant compounds from rosemary plants. J Agric Food Chem. 2002;51:375–82. 10.1021/jf025878j. 10.1021/jf025878j 12517098

[21] Mahanta BP, Bora PK, Kemprai, P, Borah, G, Lal M, Haldar S. Thermolabile essential oils, aromas and flavours: degradation pathways, effect of thermal processing and alteration of sensory quality. Food Res. Int. 2021;145. 10.1016/j.foodres.2021.110404. 10.1016/j.foodres.2021.110404 34112407

[22] Gunjal M, Khalangre A, Tosif MM, Singh J, Kaur S, Ullah R, et al. Effect of convective drying temperature and tray load density on bioactive compounds, antioxidant properties, and functional quality of radish Sango microgreens. Food Chem. 2025. 10.1016/j.foodchem.2025.144810. 40412213 10.1016/j.foodchem.2025.144810

[23] Silva MO, Castro RJS. First-order degradation kinetics of phenolic compounds and antioxidant properties of fresh and enzymatically hydrolyzed seriguela pulp (Spondias purpurea L.). ACS Food Sci Technol. 2025;5(9):3520–9. 10.1021/acsfoodscitech.5c00554.

[24] Mokrani A, Madani K. Effect of solvent, time and temperature on the extraction of phenolic compounds and antioxidant capacity of peach (*Prunus persica* L.) fruit. Sep Purif Technol. 2016;162:68–76. 10.1016/j.seppur.2016.01.043.

[25] Tourabi M, Metouekel A, Ghouizi AEL, Jeddi M, Nouioura G, Laaroussi H, et al. Efficacy of various extracting solvents on phytochemical composition, and biological properties of *Mentha longifolia* L. leaf extracts. Sci Rep. 2023;13:18028. 37865706 10.1038/s41598-023-45030-5 PMC10590439

[26] Meneses NGT, Martins S, Teixeira JA, Mussatto SI. Influence of extraction solvents on the recovery of antioxidant phenolic compounds from brewer’s spent grains. Sep Purif Technol. 2013;108:152–8. 10.1016/j.seppur.2013.02.015.

[27] Schymanski EL, Jeon J, Gulde R, Fenner K, Ruff M, Singer HP, et al. Identifying small molecules via high resolution mass spectrometry: communicating confidence. Environ Sci Technol. 2014;48:2097–8. 10.1021/es5002105. 24476540 10.1021/es5002105

[28] Bhebhe M, Füller TN, Chipurura B, Muchuweti M. Effect of solvent type on total phenolic content and free radical scavenging activity of black tea and herbal infusions. Food Anal Methods. 2016;9:1060–7. 10.1007/s12161-015-0270-z.

[29] Ogawa S, Yazaki Y. Tannins from *Acacia mearnsii* De Wild. bark: tannin determination and biological activities. Molecules. 2018;23:837. 10.3390/molecules23040837. 29621196 10.3390/molecules23040837 PMC6017853

[30] Drewes SE, Roux DG. Condensed tannins. 15. interrelationships of flavonoid components in wattle-bark extract. Biochem J. 1963;87:167–72. 10.1042/bj0870167. 16748995 10.1042/bj0870167 PMC1276856

[31] Roux DG, Paulus E. Condensed tannins. 10. isolation of (-)-butin and butein from wattle heartwoods. Biochem J. 1961;80:62–3. 10.1042/bj0800062. 13744044 10.1042/bj0800062 PMC1243951

[32] Liu W, Feng Y, Yu S, Fan Z, Li X, Li J, et al. The flavonoid biosynthesis network in plants. Int J Mol Sci. 2021;22:12824. 10.3390/ijms222312824. 34884627 10.3390/ijms222312824 PMC8657439

[33] Yu K, Song Y, Lin J, Dixon RA. The complexities of proanthocyanidin biosynthesis and its regulation in plants. Plant Commun. 2023;4:100498. 10.1016/j.xplc.2022.100498. 36435967 10.1016/j.xplc.2022.100498 PMC10030370

[34] Shen N, Wang T, Gan Q, Liu S, Wang L, Jin B. Plant flavonoids: classification, distribution, biosynthesis, and antioxidant activity. Food Chem. 2022. 10.1016/j.foodchem.2022.132531. 35413752 10.1016/j.foodchem.2022.132531

[35] Botha JJ, Ferreira D, Roux DG. Condensed tannins: direct synthesis, structure, and absolute configuration of four biflavonoids from black wattle bark (‘mimosa’) extract. J Chem Soc, Chem Commun. 1978;16:700–2. 10.1039/C39780000700.

[36] Botha JJ, Ferreira D, Roux DG. Synthesis of condensed tannins. Part 4. A direct biomimetic approach to [4,6]- and [4,8]-biflavanoids. J Chem Soc Perkin Trans 1. 1981;0:1235–45. 10.1039/P19810001235.

[37] Roux DG. The biogenesis of bark and heartwood tannins of some *Acacia* spp. and their taxonomic significance. S Afr J Sci. 1962;58:389–92.

[38] Pasch H, Pizzi A, Rode K. MALDI-TOF mass spectrometry of polyflavonoid tannins. Polymer. 2001;42:7531–9. 10.1016/S0032-3861(01)00216-6.

[39] Abilkassymova A, Aldana-Mejía JA, Katragunta K, Kozykeyeva R, Omarbekova A, Avula B, et al. Phytochemical screening using LC-MS to study antioxidant and toxicity potential of methanolic extracts of *Atraphaxis pyrifolia* Bunge. Molecules. 2024;29:4478. 10.3390/molecules29184478. 39339473 10.3390/molecules29184478 PMC11434437

