# Supplementary material for: Solvent-dependent metabolomic profiling of Acacia mearnsii bark: an untargeted metabolomics approach
Source: Anal Bioanal Chem. 2026 Apr 29;418(13):4043–67. doi: 10.1007/s00216-026-06496-0 (PMC13264538; doi:10.1007/s00216-026-06496-0)
Supplement: Supplementary file 2 — Supplementary file2 (DOCX 1.66 MB) [file 216_2026_6496_MOESM2_ESM.docx]

**Solvent-dependent Metabolomic Profiling of *Acacia mearnsii* bark: an Untargeted Metabolomics Approach**

Carolina Feistauer Gomes^a^, Giovana Domeneghini Mercali^a*^, Eliseu Rodrigues^a^

*^a^Institute of Food Science and Technology, Federal University of Rio Grande do Sul (UFRGS), Avenida Bento Gonçalves, 9500, Porto Alegre/RS 91509-900, Brazil*

**Supplementary Material**

**Contents**

**Figure S1 –** Principal components analysis (PCA) score plots of the deconvoluted feature table at MS1, including pooled quality control samples (QC): (a) score plot in negative ionization mode; (b) score plot in positive ionization mode. Extraction solvents: 50 % acetone (AC5), 70 % acetone (AC7), ambient water (AW), 50 % ethanol (E5), 70 % ethanol (E7), hot water (HW), 50 % methanol (M5), 70 % methanol (M7), quality control (QC).

**Figure S2 –** Boxplots of log-10 transformed feature abundance data: (a) negative ionization mode, and (b) positive ionization mode.

**Fig S3** Extracted ion chromatogram (EIC) of *m/z* 289 from the *Acacia mearnsii* bark extracts: (a) EIC from method A; (b) EIC from method B; and (c) EIC from method B for the evaluated samples (brown color) and for analytical standards (blue color).

**Fig S4** Fragmentation patterns of *m/z* 289 by method B for: (a) metabolite (+)-catechin, and (b) metabolite (+)-robinetinidol.

**Fig S5** Fragmentation pathway of (+)-catechin by method B.

**Fig. S6** Fragmentation pathway of (+)-robinetinidol by method B.


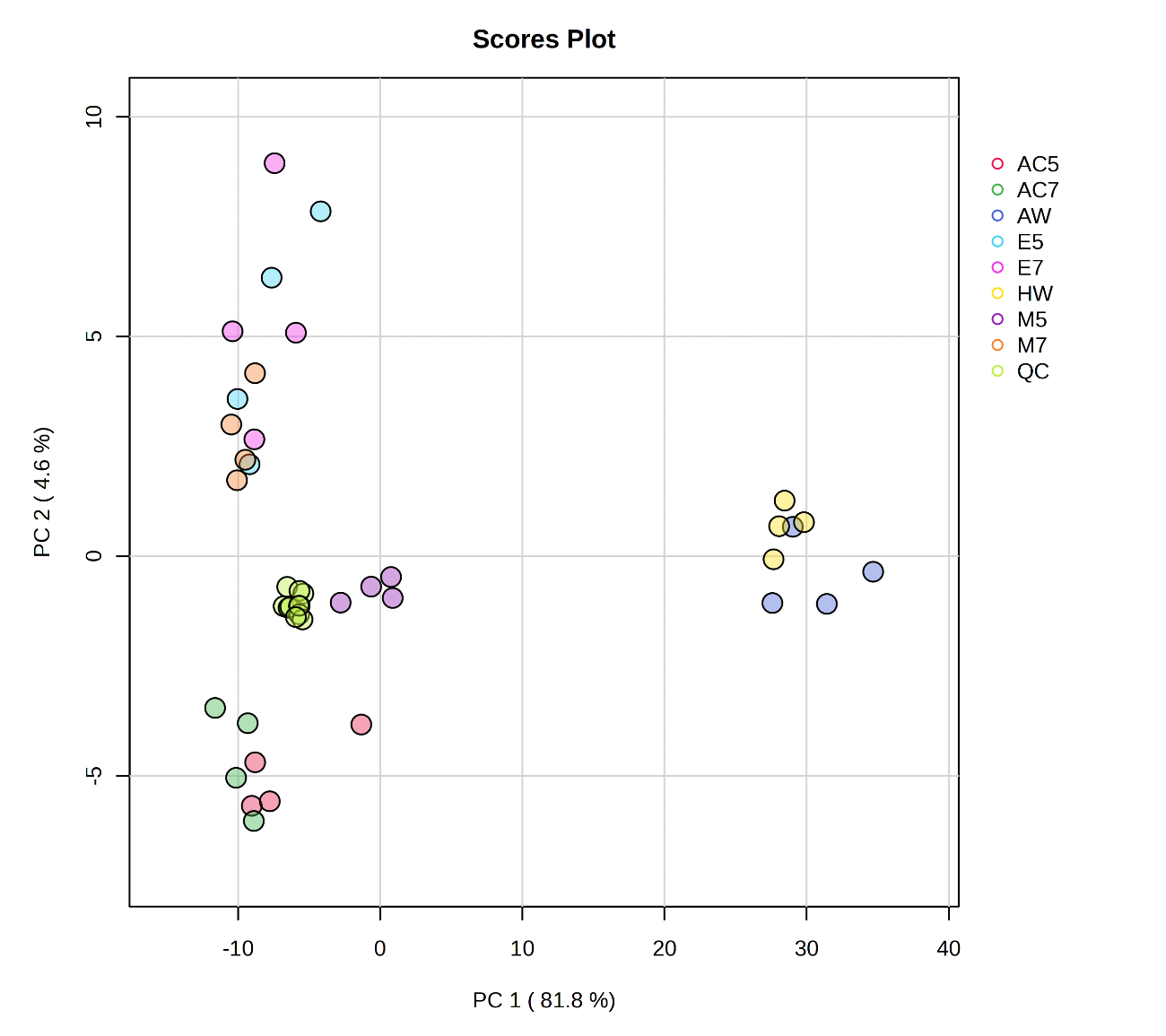

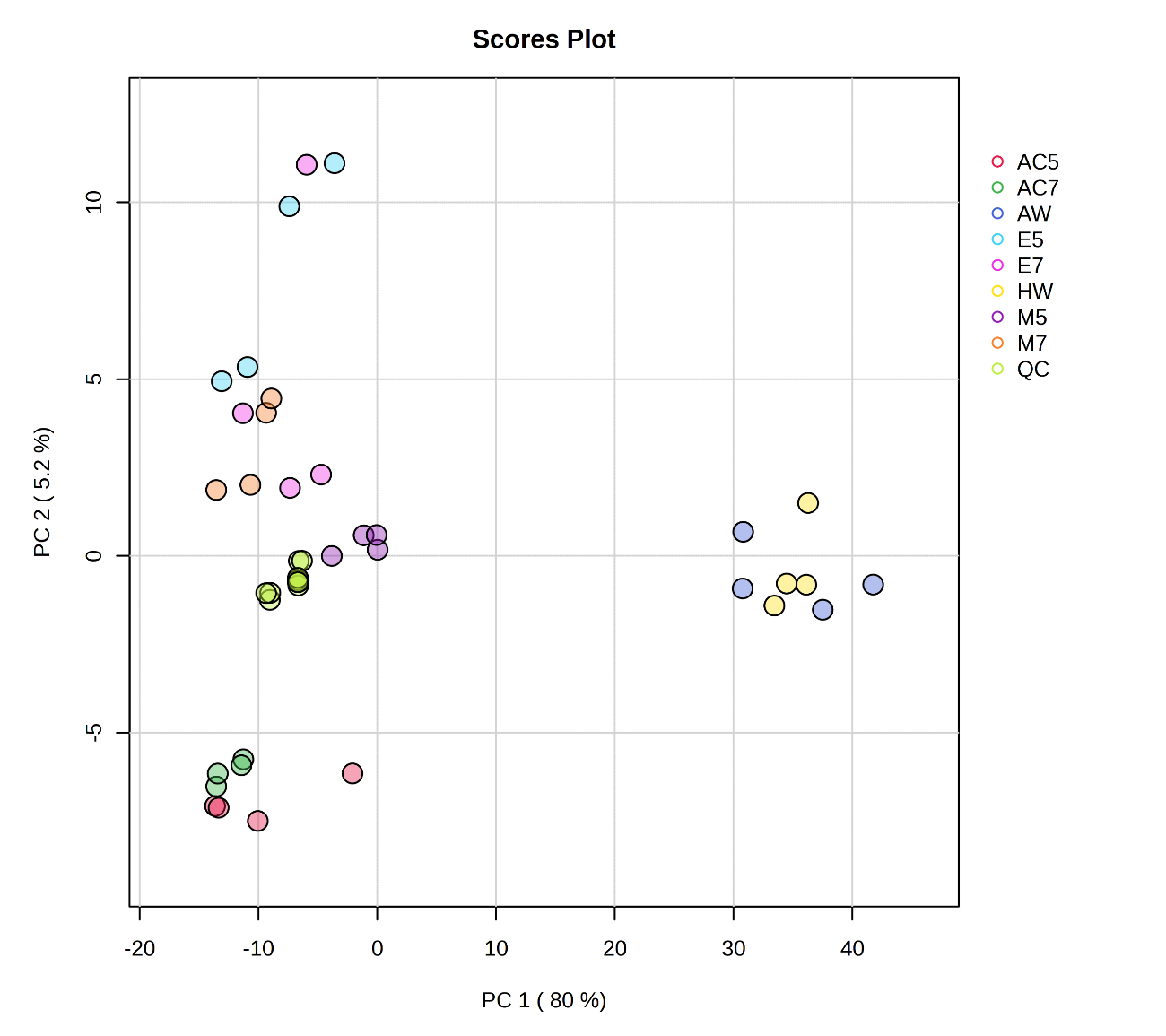


**A**

**B**

**Figure S1 –** Principal components analysis (PCA) score plots of the deconvoluted feature table at MS1, including pooled quality control samples (QC): (a) score plot in negative ionization mode; (b) score plot in positive ionization mode. Extraction solvents: 50 % acetone (AC5), 70 % acetone (AC7), ambient water (AW), 50 % ethanol (E5), 70 % ethanol (E7), hot water (HW), 50 % methanol (M5), 70 % methanol (M7), quality control (QC).

**A**

**B**


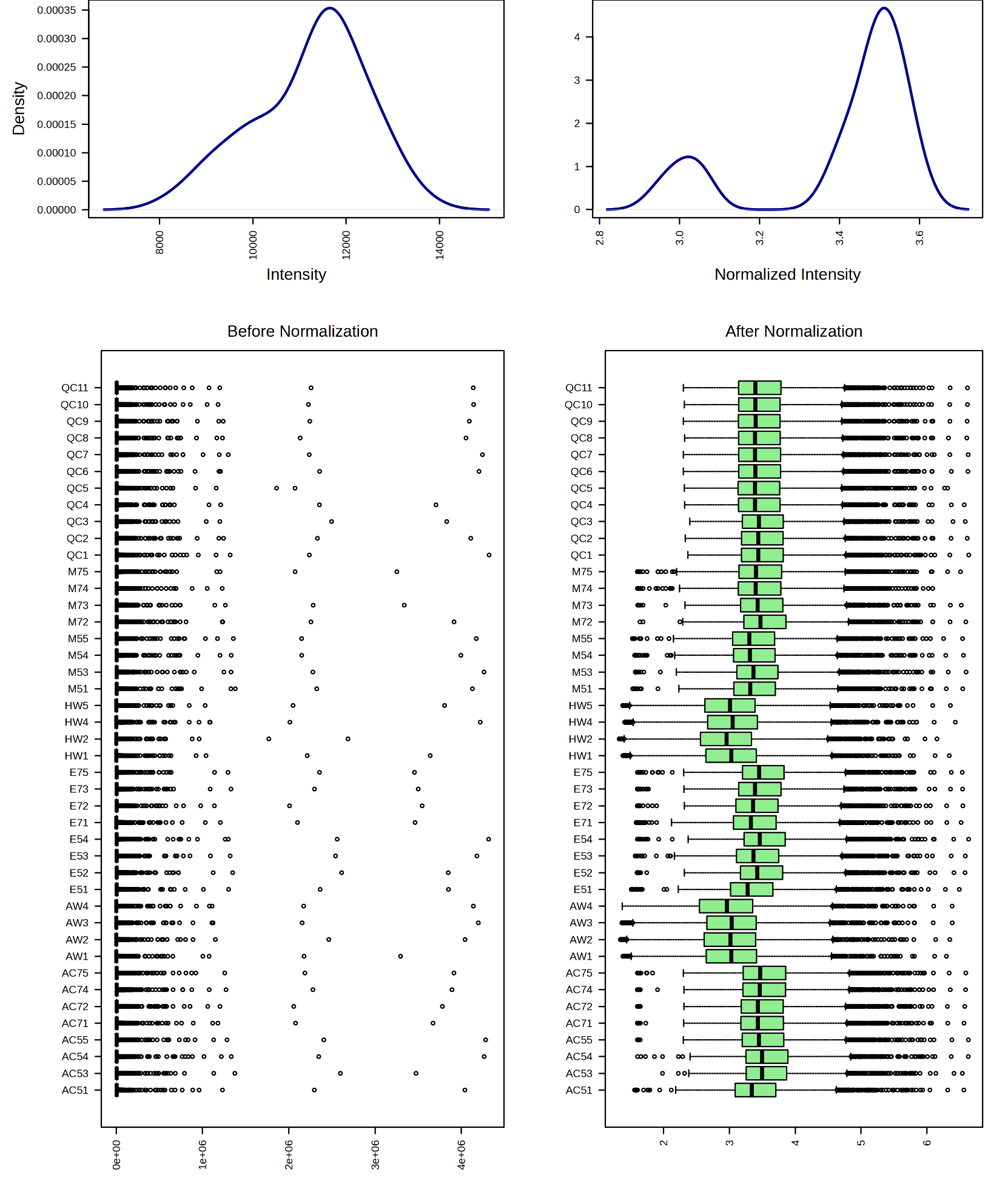


**Figure S2 –** Boxplots of log-10 transformed feature abundance data: (a) negative ionization mode, and (b) positive ionization mode.


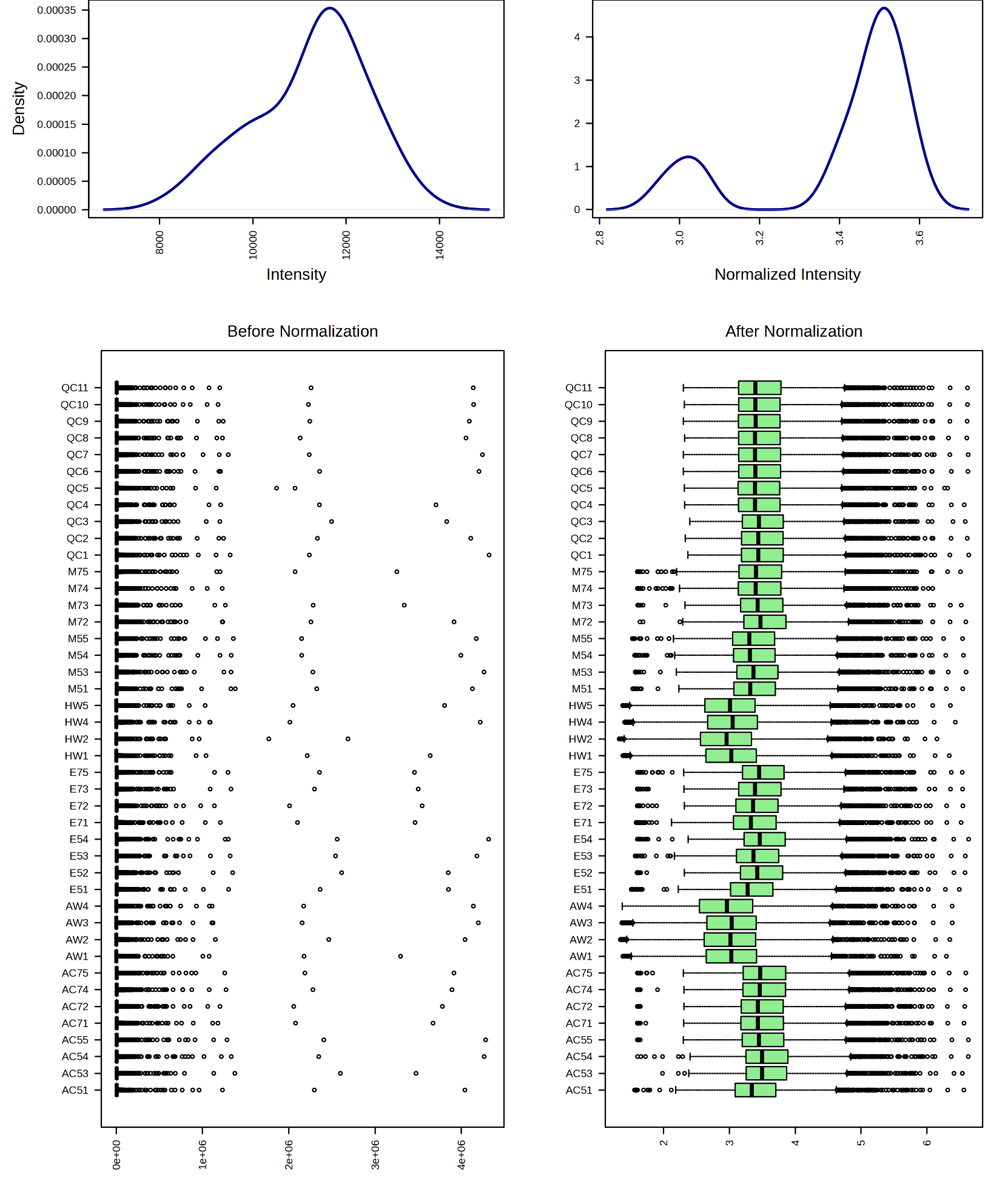


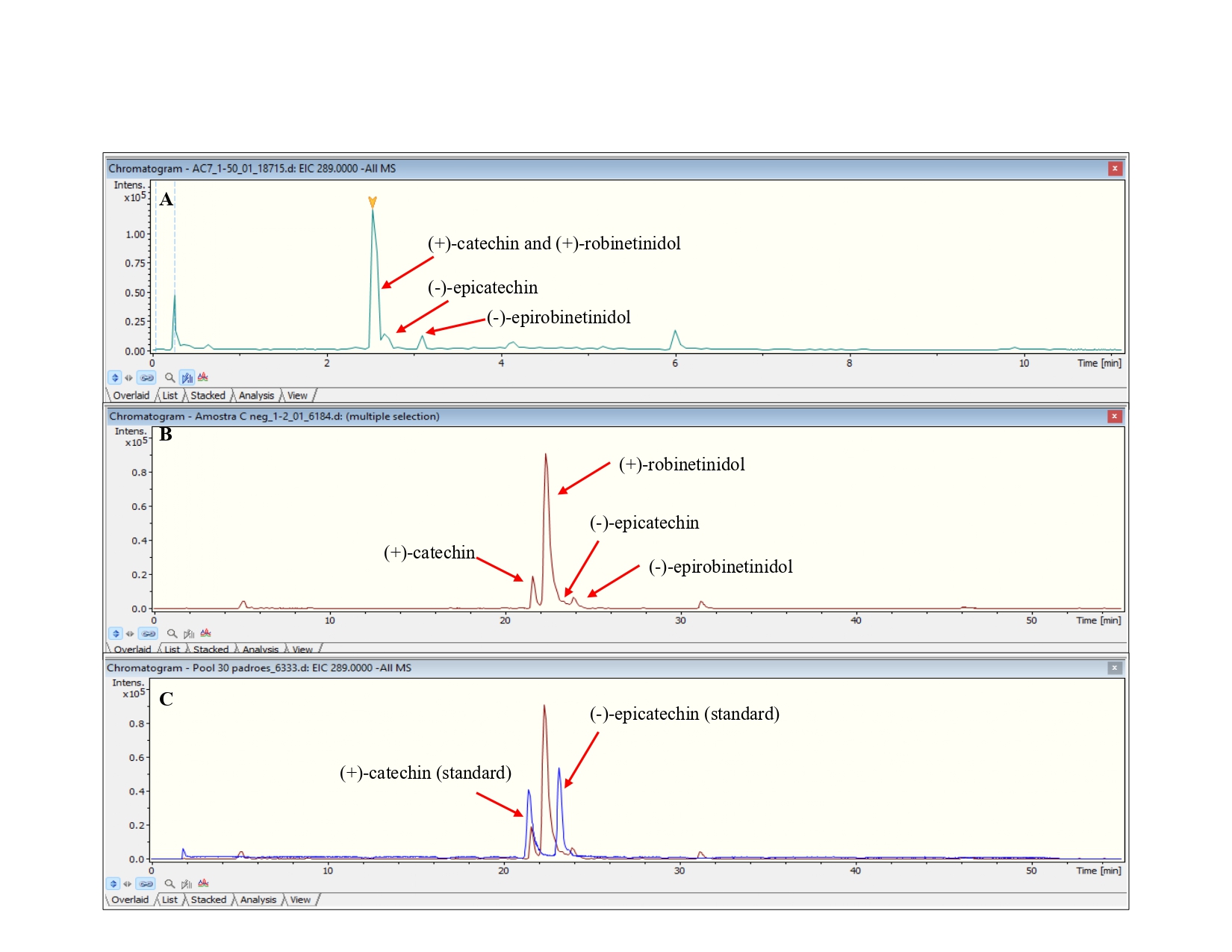
**Fig S3 –** Extracted ion chromatogram (EIC) of *m/z* 289 from the *Acacia mearnsii* bark extracts: (a) EIC from method A; (b) EIC from method B; and (c) EIC from method B for the evaluated samples (brown color) and for analytical standards (blue color).


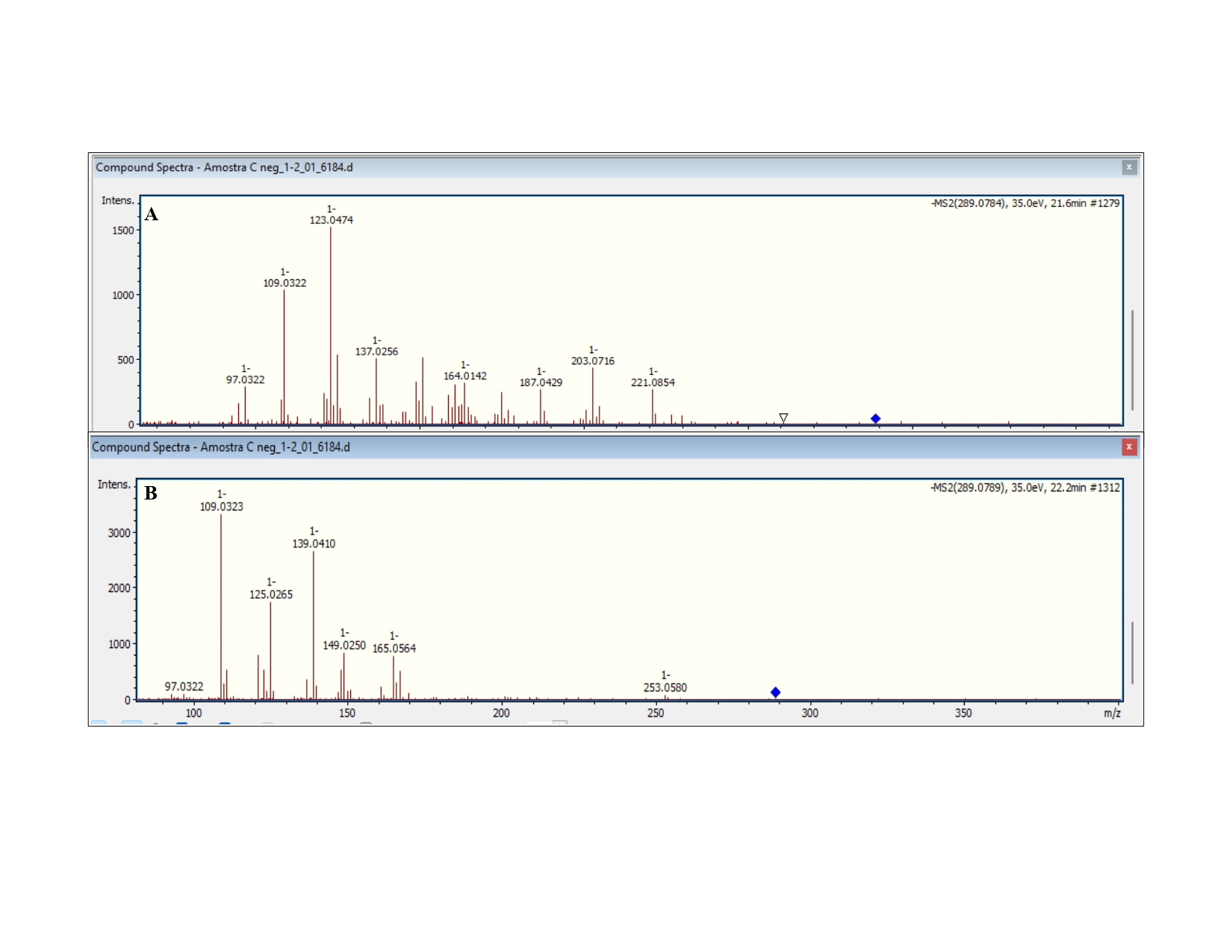
**Fig S4 –** Fragmentation patterns of *m/z* 289 by method B for: (a) metabolite (+)-catechin, and (b) metabolite (+)-robinetinidol.

**
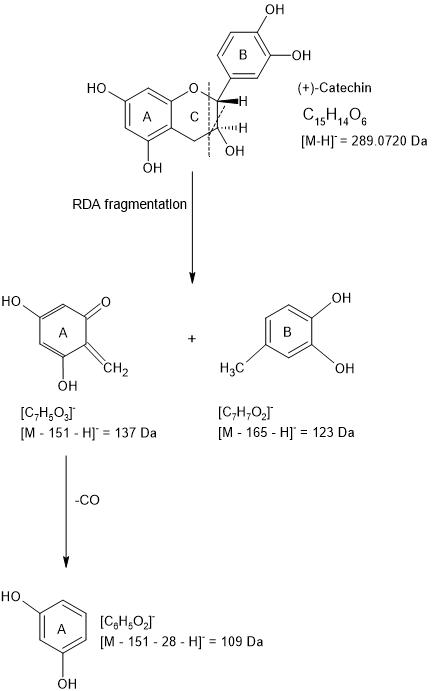
**

**Fig S5 –** Fragmentation pathway of (+)-catechin by method B.

**
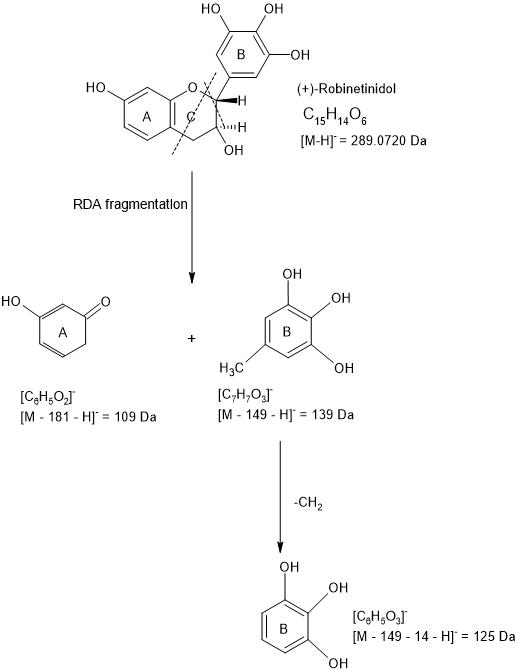
**

**Fig. S6 –** Fragmentation pathway of (+)-robinetinidol by method B.
